# Coinage Metal Compounds With 4-Methoxy-Diphenylphosphane Benzoate Ligand Inhibit Female Cancer Cell Growth

**DOI:** 10.3389/fchem.2022.924584

**Published:** 2022-07-13

**Authors:** Lorenzo Luciani, Rossana Galassi, Junbiao Wang, Cristina Marchini, Alessia Cogo, Maria Luisa Di Paolo, Lisa Dalla Via

**Affiliations:** ^1^ School of Science and Technology, University of Camerino, Camerino, Italy; ^2^ School of Biosciences and Veterinary Medicine, University of Camerino, Camerino, Italy; ^3^ Dipartimento di Scienze del Farmaco, Università degli Studi di Padova, Padova, Italy; ^4^ Dipartimento di Medicina Molecolare, Università degli Studi di Padova, Padova, Italy

**Keywords:** metal-based drug, gold, silver, copper, phosphane, breast cancer, ovarian cancer, antiproliferative activity

## Abstract

In the continuous effort to find new metal-based compounds as alternatives to platinum-related anticancer drugs, 11^th^ group metal phosphane compounds have been thoroughly taken into consideration. Tris-arylphosphane metal derivatives have been extensively considered as heteroleptic metal compounds exhibiting remarkable cytotoxic activities. Functional groups in the aryl moieties modulate the activity reinforcing or eliminating it. Previous works have highlighted that the presence of hydrophilic groups in the phosphane ligands, such as COOH or OH, hampers the anticancer activity of gold azolate/PPh_3_ compounds. To increase the polarity of the triarylphosphane ligand without affecting the activity, we considered the preparation of esters starting from the 4-diphenylphosphane-benzoic acid. The resulting phosphanes are poorer donators than the PPh_3_, leading to poly-phosphane M(I) compounds, and they exhibit intense emissive properties. A homologous series of L_3_MX-type compounds (where M = Au and X = Cl, M = Cu and X = BF_4_, and M = Ag and X = PF_6_) were obtained with the 4-methoxy-diphenylphosphane benzoate. The homologous metal compounds have been characterized by analytical and spectroscopic methods and, remarkably, their formation was associated with high frequencies of ^31^P NMR chemical shift variations (5–35 ppm in CDCl_3_). The new complexes and the ligand were evaluated on sensitive and cisplatin-resistant human tumor cell lines. The ligand is ineffective on cells while the complexes exert a notable antiproliferative effect. The homologous series of the L_3_MX complexes were able to significantly reduce the cell viability of human triple-negative breast cancer cells (MDA-MB-231), representing the most aggressive subtype of breast cancer, and of ovarian carcinoma (A2780). Among these coinage metal compounds, L_3_AgPF_6_ results the most interesting, showing the lowest GI_50_ values in all cell lines. Interestingly, this silver complex is more cytotoxic than cisplatin, taken as reference drug. The investigation of the mechanism of action of L_3_AgPF_6_ in A2780 cells highlighted the induction of the apoptotic pathway, the depolarization of the mitochondrial inner membrane, and a significant accumulation in cells.

## 1 Introduction

Cancer is one of the most occurrent leading causes of death before the age of 70 years. Among females, breast cancer is the most commonly diagnosed and the leading cause of cancer death, followed by cervical cancer, that ranks fourth for both incidence and mortality, and then ovarian cancer (Bray et al., 2018). Breast cancer appears in many subtypes and, depending on its features, a combination of surgery, radiotherapy, and platinum-based chemotherapy (Schwentner et al., 2011; Wang et al., 2016) is applied for the treatment. As concerning ovarian cancer, it is noteworthy that every year there are over 295,000 new diagnoses and over 184,000 deaths globally (Bray et al., 2018). After cytoreductive surgery, all patients generally receive platinum-based therapy with either cisplatin or carboplatin in combination with alkaloid as taxanes (Jelovac and Armstrong, 2011). In both breast and ovarian cancers, the above chemotherapy treatments improve the overall survival of the patient, but the occurrence of drug resistance and/or serious side effects often compromise long-term effectiveness and most patients eventually relapse. In this regard, the research of the alternative anticancer agent is an open issue. Metal-based drugs, with respect to the purely organic molecules, afford additional effects as the result of protein or DNA bindings by the metal moieties (Anthony et al., 2020). In this regard, it is steadily rising up the production of data, supporting the evidence that coinage metal complexes may represent a valid alternative to the classic chemotherapy in very aggressive tumors (Tan et al., 2010; Biersack et al., 2012; Petanidis et al., 2017; Yeo et al., 2018; Boros et al., 2020). Gold phosphane compounds have received a great interest after the observation that patients under chrysotherapy treatment for rheumatoid arthritis were immune to the onset of tumors and/or inflammatory diseases (Park et al., 2014). From this early observation, there has been considerable effort in both the design and testing of Au(I) phosphine compounds, attempting to grasp the relationship between the structure of the compounds and their activity. The lipophilicity of the ligand is recognized to be the key point for the cytotoxic activity of arylphosphane compounds based on coinage metals (Dominelli et al., 2018; Mirzadeh et al., 2019). A key step of the gold compound action is the coordination of Cys-34 of serum albumin in physiological media (Sadler and Sue, 1994), which acts as both drug scavenger and/or transporter. The phosphorous–gold bond possesses a rather high thermodynamic stability, which is comparable to that of sulfur of Cys-34, avoiding a fast and complete abrogation of the effect of gold-based drugs (Berners-Price and Filipovska, 2011; Meyer et al., 2012). Additionally, it is recognized that the mechanism of action for gold-based compounds passes through the covalent bonding to thioredoxin reductase, TrxR (Berners-Price et al., 1999). Less attention has been given to silver compounds, but remarkable activities have been found for several Ag(I) phosphanes in many cancer cell lines even cisplatin-resistant (Zartilas et al., 2009; Hecel et al., 2019). Polyphosphane copper compounds with the CuP_4_ core have been studied on many cancer cells showing that the kinetic inertness does not correspond to higher activity, hence a certain lability of the ligand seems to promote cytotoxicity (Santini et al., 2014). Moreover, *in vitro* antitumor activity of the water-soluble copper(I) complexes bearing the tris(hydroxymethyl)phosphine ligand has been ascertained (Marzano et al., 2008).

However, in a homologous series of complexes, the coordination ability, the strength of the metal phosphorus bonds, and the tendency to hydrolyze or to respond to the redox environment of the copper, silver, and gold phosphane compounds are rather different (Kaim et al., 2013). Basically, as an effect of the diverse multiple events imputed to the presence of different metals in the complexes, similar structures may result in completely different biological activity outputs. Moreover, in order to consider fragment-based drug discovery (FBDD) approaches (Morrison et al., 2020), in addition to the ability to make hydrogen bonding, it is debated that the 3D fragments are largely preferable because molecular shape is one of the most important factors ruling the molecular recognition of a biomolecule (Morrison et al., 2020). To approach this aim, we considered the preparation of methyl ester derivatives of the 4-diphenylphosphane benzoic ligand (L^OMe^) to obtain a homologous series of poly-phosphane coinage metal compounds having tri- or tetracoordinated metal fragments. Previous preparative studies (Pucciarelli et al., 2019) highlighted the tendency of L^OMe^ to form rather stable poly-phosphane complexes even for gold, which forms preferentially linear mono- or bis-phosphane compounds. However, in earlier studies, it was ascertained that the presence of the polar protic groups in the ligands hampers the anticancer activity in breast cancer cells (Gambini et al., 2018; Galassi et al., 2021); therefore, in this work, we prepared the homologous series of tris-phosphane complexes with the ester derivative, L^OMe^, and the coinage metals in the +1 oxidation state to evaluate the antiproliferative effect toward a panel of human tumor cell lines and female tumors, such as breast cancer (MDA-MB231 cells) and ovarian carcinoma pairs A2780 and A2780cis, sensitive and resistant to cisplatin, respectively. Biological investigations were performed to elucidate the mechanism of action of the most active complex.

## 2 Materials and Methods

### 2.1 Syntheses and Characterization

#### 2.1.1 Materials

4-Diphenylphosphine benzoic acid and other chemicals were purchased from Merck and used without any other purifications. A foil of metal gold was used to synthesize tetrachloride gold(I) acid by dissolving the gold chop by boiling aqua regia and by the careful evaporation of water till almost to dryness. Me_2_SAuCl was prepared by the reduction of tetrachloride gold(I) acid with an excess of Me_2_S in ethyl alcohol. The solvents used in the preparations were HPLC grade and they were used as purchased. Anhydrous and radical-free THF was obtained by treating the solvent with Na/acetophenone under a N_2_ atmosphere.

#### 2.1.2 Characterization

Elemental analyses (C, H, N, and S) were performed in-house with a Fisons Instruments 1108 CHNS-O Elemental Analyser. Melting points were taken on an SMP3 Stuart.

#### 2.1.3 Scientific Instruments

IR spectra were recorded from 4,000 to 100 cm^−1^ with a Perkin-Elmer SPECTRUM ONE System FT-IR instrument. IR annotations used: br = broad, m = medium, s = strong, sh = shoulder, vs. = very strong, w = weak, and vw = very weak. ^1^H and ^31^P NMR spectra were recorded on an Oxford-400 Varian spectrometer (400.4 MHz for ^1^H and 162.1 MHz for ^31^P). Chemical shifts, in ppm, for ^1^H NMR spectra are relative to internal Me_4_Si. ^31^P NMR chemical shifts were referenced to an 85% H_3_PO_4_ standard. The ^31^P NMR spectroscopic data were accumulated with ^1^H decoupling. NMR annotations used: br = broad, d = doublet, dd = double doublet, t = triplet, m = multiplet, s = singlet. Electrospray mass spectra (ESI-MS) were obtained in a positive- or negative-ion mode on a Series 1100 MSD detector HP spectrometer, using an acetonitrile or methanol mobile phase. The compounds were added to reagent-grade acetonitrile to give solutions of approximate concentration 0.1 mM. These solutions were injected (1 µL) into the spectrometer *via* a HPLC HP 1090 Series II fitted with an auto-sampler. The pump delivered the solutions to the mass spectrometer source at a flow rate of 300 μL min^−1^, and nitrogen was employed as both a drying and nebulizing gas. Capillary voltages were typically 4000 and 3500 V for the positive- and negative-ion modes, respectively. Confirmation of all major species in this ESI-MS study was aided by comparison of the observed and predicted isotope distribution patterns, the latter calculated using the IsoPro 3.0 computer program.

#### 2.1.4 Preparations

##### 2.1.4.1 Preparation of the L^OMe^

To a solution of 4-diphenylphosphanyl-benzoic acid (398 mg; 1.30 mmol) in 15 mL of anhydrous CH_2_Cl_2_, methanol (0.05 mL; 1.4 mmol) to obtain L^MeO^ and N,N-dimethyl-amino-pyridine (37.0 mg; 0.30 mmol) were added. To this solution, N,N-diisopropylcarbodiimide (0.22 mL; 1.4 mmol) dissolved in 3 mL of anhydrous CH_2_Cl_2_ was added dropwise in 15 min, maintaining the solution at 0°C. The resulting yellow suspension was allowed to stir for 16 h at room temperature. The suspension was filtered and evaporated to dryness. The recovered solid was dissolved in 30 mL of ethyl acetate, washed with a watery solution at 10% of HCl (15 mL), a saturated solution of NaHCO_3_ (15 mL), and brine (15 mL). The organic fraction was recovered and treated with solid anhydrous Na_2_SO_4_, filtered, and evaporated to dryness. The raw material was purified as a waxy solid by flash chromatography by eluting with a mixture of 5% EtOAc 95% hexane. Yield: 76%. M. p. 97–99°C.

^1^H NMR (CDCl_3_, δ): 8.05 (d, 2H); 7.35–7.40 (m, 12 H); 3.95 (s, 3H).

^31^P NMR (CDCl_3_, δ): −5.02 (s).

MIR (cm^−1^): 3067 (w), 2999 (w), 2952 (w), 2924 (w), 2852 (w), 2102 (w), 1943 (w), 1822 (w), 1719 (vs), 1597 (m, sh), 1584 (sh), 1561 (w), 1476 (m), 1433 (s), 1393 (m), 1278 (vs), 1181 (m), 1115 (m), 1088 (s), 1017 (s), 999 (sh), 965 (m), 857 (m), 750 (m), 743 (vs).

FIR (cm^−1^) 693 (s), 670 (sh, m), 634 (w), 618 (w), 530 (m), 497 (s), 485 (s), 462 (sh, m), 431 (m), 410 (w), 396 (w), 346 (m), 303 (w), 287 (m), 280 (sh, m), 250 (m), 239 (sh, w), 226 (sh, w), 211 (m), 194 (w), 174 (m), 165 (w), 152 (w), 142 (w), 131 (w), 127 (w), 117 (w), 112 (w), 105 (w).

ESI (+) (CH_3_CN, m/z): 321 (100) [L^OMe^ + H]^+^.

Elemental analysis calculated for C_20_H_17_O_2_P, (%): C 74.99, H 5.35. Found C 75.5, H 4.69:

$${\left(L^{\mathrm{OMe}}\right)}_{3}\mathrm{CuB}F_{4},\text{(1)}$$

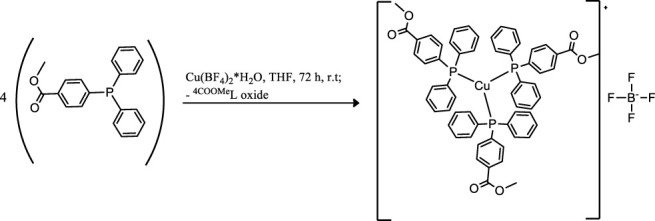


The solution of L^OMe^ (100 mg; 0.312 mmol) in anhydrous THF (6 mL) was added dropwise to the solution of copper tetrafluoroborate hexahydrate (27 mg; 0.078 mmol) in THF (4 mL) at 0°C. The mixture was stirred at r. t. for 72 h, then filtered and dried at reduced pressure. The white solid was washed in hexane (3 × 4 mL) to remove the phosphine oxide byproduct. The suspension was centrifuged, and the solid was dried under reduced pressure to obtain a white powder. Yield: 75%.

^1^H-NMR (CDCl_3_, δ, 293K): 7.74 (d, ^3^J_H-H_ = 8 Hz, 6H); 7.40 (m, 6H), 7.24-7.12 (m, 30H), 3.93 (s, 9H).

^31^P-NMR (CDCl_3_, δ, 293K): 0.30 (s, br).

^1^H-NMR (acetone-d^6^, δ, 293K): 7.87 (d, ^3^J_H-H_ = 8 Hz, 6H); 7.55 (m, 6H), 7.40-7.31 (m, 30H), 3.91 (s, 9H).

^31^P-NMR (acetone-d^6^, δ, 293K): -0.45 (s, br).

MIR (cm^−1^): 3074 (w, sh), 3057 (w), 3007 (w), 2954 (w), 2926 (w), 2852 (w), 1721 (vs), 1599 (m), 1562 (w, sh), 1483 (m), 1436 (s), 1395 (m), 1313 (m, sh), 1280 (vs), 1217 (w), 1187 (m), 1161 (m, sh), 1115 (sh, s), 1090 (vs, br), 1071 (s, sh), 1063 (s), 1017 (s), 998 (m), 963 (w, sh), 907 (m) 854 (m, sh), 828 (m), 804 (m), 761 (m, sh), 746 (m), 733 (s), 723 (s).

FIR (cm^−1^): 693 (s), 665 (w, sh), 633 (w), 617 (w), 593 (w), 563 (m), 540 (m), 531 (m, sh), 509 (s), 485 (m, sh), 458 (m), 442 (m), 423 (m), 405 (w), 396 (w), 361 (m, sh), 340 (m), 303 (m, sh), 294 (m), 279 (m), 267 (w), 256 (w), 249 (w), 235 (w), 226 (w), 214 (w), 207 (w), 198 (w), 185 (m), 173 (m), 166 (m), 155 (m), 142 (m), 132 (w), 125 (m, sh), 120 (s), 102 (w).

ESI (+) (CH_3_OH, m/z): 1023 (14) [(L^OMe^)_3_Cu]^+^, 703 (100) [(L^OMe^)_2_Cu]^+^.

ESI (-) CH_3_OH, m/z): 197 (100).

Elemental analysis calculated for C_60_H_51_BcuF_4_O_6_P_3_, (%): C 64.85, H 4.63. Found C 65.02, H 4.69:

$${\left(L^{\mathrm{OMe}}\right)}_{3}\mathrm{AgP}F_{6},\text{(2)}$$

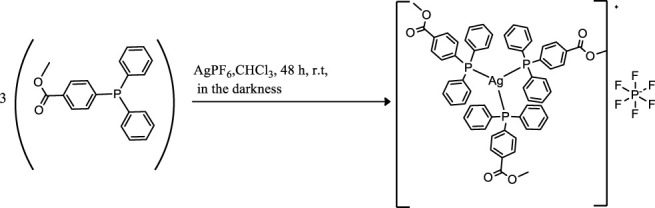


The solution of L^OMe^ (100 mg; 0.312 mmol) in 3 mL CHCl_3_ was added dropwise to the solution of silver hexafluorophosphate (26 mg; 0.104 mmol) in CHCl_3_ (3 mL) at 0 °C. The mixture was stirred at r. t. for 48 h, then filtered and dried at reduced pressure. The white solid was washed with hexane (3 × 5 mL) and dried at reduced pressure. Yield: 90%.

^1^H-NMR (acetone-d^6^, δ, 293K): 7.84 (d, ^3^J_H-H_ = 8 Hz, 6H); 7.44-7.39 (m, 6H), 7.30-7.24 (m, 30H), 3.91 (s, 9H).

^31^P-NMR (acetone-d^6^, δ, 293K): 7.67 (s, br), -144.25 (spt).

^31^P-NMR (CDCl_3_, δ, 293K): 5.49 (br), -18.56 (t, ^1^J_P-F_ = 973.81 Hz, PO_2_F_2_^−^), -144.25 (spt, ^1^J_P-F_ = 715 Hz,PF_6_).

MIR (cm^−1^): 3076 (w, sh), 3056 (w), 3006 (w), 2954 (w), 2847 (w), 1722 (vs), 1598 (m), 1586 (w, sh), 1562 (w), 1495 (w, sh) 1482 (m), 1462 (w, sh), 1456 (w, sh), 1436 (s), 1394 (m), 1311 (m, sh), 1280 (vs), 1187 (m), 1160 (m, sh), 1118 (m), 1112 (s), 1091 (s), 1017 (m), 1000 (m), 963 (m), 921 (w, sh), 876 (m, sh), 837 (vs), 761 (m, sh), 744 (vs), 722(s).

FIR (cm^−1^): 693 (s), 675 (m, sh), 665 (w, sh), 644 (w), 633 (w), 618 (w), 586 (w), 565 (w), 556 (m), 529 (m, sh), 506 (s), 484 (s), 467 (m, sh), 459 (m), 445 (m), 437 (m), 400 (m), 379 (m), 352 (m, sh), 338 (m), 317 (m, sh), 311 (w), 297 (m), 287 (w), 279 (w), 267 (w), 250 (w), 237 (w), 223 (m), 208 (w), 197 (w), 193 (w), 184 (w), 174 (m), 167 (m), 156 (m), 151 (m), 142 (m), 122 (s), 115 (m, sh), 103 (m).

ESI (+) (CH_3_OH, m/z): 747 (100) [((L^OMe^)_2_Ag)]^+^, 393 (11), 360 (23).

ESI (-) (CH_3_OH, m/z): 145 (100) [PF_6_^−^], 127 (95) [PF_5_H^−^], 101 (45) [PO_2_F_2_^−^].

Elemental analysis calculated for C_60_H_51_AgF_6_O_6_P_4_, (%): C 59.37, H 4.24. Found (%): C 59.09, H 4.24:

$${\left(L^{\mathrm{OMe}}\right)}_{3}\mathrm{AuC}l,\text{(3)}$$

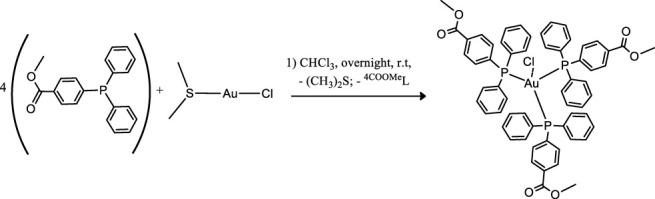


The solution of L^OMe^ (64 mg; 0.2 mmol) in anhydrous CHCl_3_ (2 mL) was added dropwise to the solution of (dimethylsulfide)gold(I)chloride (15 mg; 0.05 mmol) in CHCl_3_ (3 mL) at 0°C. The mixture was stirred at r. t. overnight, then filtered and dried at reduced pressure. The pale-yellow solid was washed with hexane (3 × 5 mL) to remove the dimethylsulfide and the excess of the L^OMe^ ligand; the ivory precipitate was dried under vacuum. Yield: 70%.

^1^H-NMR (CDCl_3_): 7.97 (dd, ^3^J_H-H_ = 8 Hz, 2 Hz, 6H); 7.46–7.36 (m, 36H), 3.95 (s, 9H).

^31^P-NMR (CDCl_3_): 30.57 (s).

^31^P-NMR (acetone-d^6^, δ, 293K): 24.08 (s), 8.58 (br).

^31^P-NMR (acetone-d^6^, δ, 193K): 24.83 (s).

MIR (cm^−1^): 3055 (w), 3007 (w), 2952 (w), 2844 (w), 1720 (vs), 1599 (m), 1562 (m), 1483 (w), 1435 (s), 1393 (m), 1313 (sh, w), 1282 (vs), 1273 (vs), 1191 (m), 1119 (sh, m), 1096 (s), 1018 (m), 998 (m), 961 (w), 856 (m), 829 (w), 804 (w), 762 (m, sh), 763 (s), 751 (s), 728 (s), 711 (sh, m).

FIR (cm^−1^): 692 (vs), 632 (w), 618 (w), 589 (w), 556 (m), 525 (m), 510 (s), 485 (m), 456 (m), 443 (m), 428 (m), 400 (w), 367 (w), 333 (s), 326 (sh, s), 300 (m), 285 (w), 278 (w), 273 (w), 267 (w), 248 (m), 237 (w), 226 (m), 214 (w), 207 (m), 194 (m), 185 (m), 172 (m), 165 (m), 161 (m), 151 (m), 143 (m), 132 (m), 120 (m), 114 (m), 101 (w).

ESI (+) (CH_3_OH, m/z, relative intensity): 837 (100) [(L^OMe^)_2_Au]^+^.

Elemental analysis calculated for C_60_H_51_AuClO_6_P_3_ (%): C 60.39, H 4.31. Found (%): C 60.15, H 4.47.

### 2.2 Biological Assays

#### 2.2.1 Cell Cultures

HT-29 (colorectal cancer, ATCC, United States), H1975 (non-small-cell lung cancer, ATCC, United States), A2780 (human ovarian carcinoma, ECACC, United Kingdom), and A2780cis (human ovarian carcinoma cisplatin-resistant, ECACC, United Kindom) were grown in RPMI 1640 (Sigma Chemical Co. R6504). 1.5 g/L NaHCO_3_, 10% heat-inactivated fetal bovine serum (Biowest), 100 U/mL penicillin, 100 μg/mL streptomycin, and 0.25 μg/mL amphotericin B (Sigma Chemical Co. A5955) were added to the medium. A sub-lethal dose of 1 μM cisplatin was added to A2780cis every 3–4 passages to maintain the drug resistance. Human MDA-MB-231 cells represent an *in vitro* model of triple-negative breast cancer since they do not express estrogen receptor, progesterone receptor, and HER2 (human epidermal growth factor receptor 2) (ER-/PR-/HER2-). They were obtained from American Type Culture Collection (Rockville, MD) and cultured in Dulbecco’s modified essential medium (DMEM, Gibco, Life Technologies) supplemented with 10% fetal bovine serum (FBS, Gibco, Life Technologies) and 1% penicillin–streptomycin (Gibco, Life Technologies). Cells were cultured in standard conditions at 37°C under a humidified atmosphere with 5% CO_2_.

#### 2.2.2 Inhibition Growth Assay

HT-29, H1975, A2780, and A2780cis cells (5 × 10^4^) were seeded into each well of a 24-well cell culture plate. After incubation for 24 h, different concentrations (from 0.1 to 20 μM) of the test complexes were added to the complete medium and cells were incubated for a further 48 h. The cells were detached using trypsin/EDTA, stained with Trypan blue dye, and counted in a Burker chamber. The percentage of unstained viable cells over the total number of cells was calculated for each experimental set. Data are the mean (±SD) of at least three independent experiments in duplicate. Cytotoxicity data were expressed as GI_50_ values, i.e., the concentration of the test agent inducing 50% reduction in cell number compared with control cultures. Stock solutions (20 mM) of the metal complexes were prepared by dissolving weighed amounts of the solid in dimethylsulfoxide, maintained in the dark at 0°C, and used within 2 weeks. Working solutions of the appropriate complex concentration were prepared by dilution of the stock solutions with complete medium in such a way that the final amount of solvent in each well did not exceed 0.5%. Cisplatin was dissolved in 0.9% NaCl at a 4 mM concentration.

The effects of compounds **1**, **2**, and **3** on the MDA-MB-231 cell viability were evaluated by seeding 1 × 10^4^ cells/well in 96-well plates in complete medium (DMEM supplemented with 10% FBS). The day after, fresh medium containing increasing concentrations of each compound ranging from 0.1 to 100 μM was added. Cell viability was determined after 24 h or 48 h using an MTT [3-(4,5-dimethylthiazol-2-yl)-2,5-diphenyl-2H-tetrazolium bromide, Sigma Aldrich, St. Louis, MO] assay, which is based on the conversion of MTT to formazan crystals by mitochondrial enzymes. The formazan deposits were dissolved in DMSO, and the absorbance of each well was measured at 540 nm in a Multiskan Ascent 96/384 Plate Reader. The cytotoxicity of the compounds was expressed as a percentage of viable cells relative to control cells (vehicle alone). Each drug concentration was evaluated with six replicates, and each experiment was repeated three times. At the end of the experiments, a dose–response curve was plotted, and the GI_50_ concentrations were determined.

Statistical analysis was performed using One-Way ANOVA analysis of variance followed by Dunnett’s multiple comparison test. Quantitative data are presented as means ± standard deviation (SD). *p* ≤ 0.05 was used as the critical level of significance.

#### 2.3.3 Apoptotic Cell Death Analysis

The apoptosis process was assayed by using the FITC Annexin V Apoptosis Detection Kit I (BD Pharmigen). A2780 cells (2.0 × 10^5^) were seeded into culture dishes in complete growth medium and allowed to grow in standard conditions for 24 h. The test complex was then added at 0.1, 0.2, and 0.3 μM concentrations, and cells were incubated for a further 48 h. After treatment, cells were collected and washed twice with phosphate-buffered saline (8 mM Na_2_HPO_4_⋅2H_2_O, 1.5 mM KH_2_PO_4_, 2 mM KCl, 0.1 M NaCl, PBS). A suspension of 10^6^ cells/mL in binding buffer (100 μL) was added with Annexin V-FITC and propidium iodide (PI), as indicated by the supplier’s instructions, and incubated for 15 min at room temperature in the dark. Finally, a volume of binding buffer to reach a final volume of 600 μL was added. The populations of Annexin V-negative/PI-negative (viable), Annexin V-positive/PI-negative (early apoptosis), Annexin V-positive/PI-positive (late apoptosis), and Annexin V-negative/PI-positive (necrosis) were detected by the FACSAria III flow cytometer (Becton–Dickinson, Mountain View, CA), and data were analyzed by BD FACSDiva software (Becton–Dickinson).

#### 2.3.4 Cell Cycle Distribution

A2780 cells (2 × 10^5^) were seeded in culture dishes with complete medium and incubated at 37°C in a 5% CO_2_ humidified atmosphere. After 24 h, the cells were treated with the test compound at 0.1, 0.2, and 0.3 μM concentrations and incubated for 48 h in standard conditions. Cells were then harvested, centrifuged, and 3 × 10^5^ cells for each sample were treated with ice-cold 70% w/v ethanol at 4°C for 20 min. Then, cells were washed twice with PBS and resuspended in a final volume of 300 μL PBS containing 0.1 mg mL^−1^ RNAse (Merck R6513) and 36 μg mL^−1^ PI (Merck P4170). The analysis of the DNA content was performed by the FACSAria III flow cytometer, and data were analyzed by BD FACSDiva software.

#### 2.3.5 Mitochondrial Transmembrane Potential Measurement

Mitochondrial transmembrane potential was assayed in A2780 cells by the BD™ MitoScreen Kit (BD Pharmigen). Cells (2 × 10^5^) were seeded in culture dishes with complete medium and incubated for 24 h in standard conditions. The test complex was added at 0.1, 0.2, and 0.3 μM concentrations, and drug-treated cells were incubated for a further 48 h. The cells were then harvested, resuspended (3 × 10^5^ cells) in the JC-1 Working Solution, and incubated for 30 min at 37°C in the dark. Following incubation, cells were washed twice with PBS, resuspended, and immediately analyzed by the FACSAria III flow cytometer. Data were analyzed by BD FACSDiva software.

#### 2.3.6 Glutathione and Total Thiol Assay

A2780 cells (5 × 10^5^) were incubated with a test compound at a 0.2 μM concentration for 48 h. Then, cells were collected and the pellet was frozen in liquid nitrogen and stored at −78°C until use.

Analysis of reduced glutathione (GSH) and glutathione disulfide (GSSG) were performed as previously reported (Giustarini et al., 2013) with some modifications (Gentilin et al., 2021).

Briefly, pellets were quickly thawed and lysed with ice-cold buffer (50 mM tris/HCl, containing 10 mM boric acid, 1 mM serine, 1 mM EDTA, and protease inhibitor cocktail, pH 8) at a concentration of 40 × 10^6^ cell/mL. To assay the total glutathione (GSH + GSSG) content, lysate was added with 60% trichloroacetic acid (TCA) to obtain a 7.5% final concentration, centrifuged at 14,000 g for 5 min at 4°C, and the supernatant was immediately used for the spectrophotometric glutathione reductase recycling assay. For the GSSG analysis, 1-methyl-2-vinylpyridinium triflate (M2VP, 3.5 mM, final concentration) was used as a thiol-masking reagent (Shaik and Mehvar, 2006). After 3 min of incubation with M2PV at room temperature, the samples were centrifuged at 14,000 g for 5 min at 4°C. The supernatant was kept in ice until the GSSG assay. The total RSH was determined by measuring the reduction of 5,5’-dithiobis (2-nitrobenzoic acid) (DTNB) to 2-nitro-5-thiobenzoate ion (TNB) at 412 nm (ε_412 nm_ = 13,640 M^−1^ cm^−1^, DTNB 100 μM in 200 mM potassium phosphate/EDTA 1 mM, pH 7.4).

GS_tot_ and GSSG concentrations were determined according to the spectrophotometric recycling procedure, in the DTNB assay buffer, in the presence of glutathione reductase 0.4 U/mL (final concentrations) and NADPH (100 μM) (Shaik and Mehvar, 2006). Spectrophotometric analysis was performed by a Varian Cary 50 UV–Vis spectrophotometer (Agilent Technologies, Milan, Italy). The amount of GSH was then calculated by subtracting GSSG (multiplied by 2) from the levels of total glutathione (GS_tot_). The GS_tot_ and GSSG concentrations in the unknown sample were calculated by using standard calibration curves. The calculated glutathione and thiol concentrations were normalized to the protein concentration determined according to Bradford, using bovine serum albumin as the protein standard (Bradford, 1976). All assays were performed at least in triplicate. Analysis of data and *t*-test were performed using the Sigma Plot software, version 10.0 (Jandel Scientific, San Rafael, CA, United States).

#### 2.3.7 Cell Uptake

A2780 cells (2 × 10^6^) were seeded into each cell culture plate in complete growth medium, allowed to grow for 24 h, and then the test complex was added at a 75 μM concentration for different incubation times. Cells were harvested, the pellet was resuspended in 2 mL of 0.9% NaCl, and centrifuged at 12000 g. The pellet was washed twice with 0.9% NaCl and mineralized in a digital dry bath at 90°C for 1 h in 390 μL of HNO_3_ (65%). Finally, the samples were diluted up to 5 mL in milliQ^®^ water.

The content of P, Ag, and Au was analyzed by a Spectroflame Modula sequential and simultaneous ICP-spectrometer (ICP SPECTRO Arcos with EndOnPlasma torch) equipped with a capillary cross-flow Meinhard nebulizer (Spectro Analytical Instruments, Kleve, Germany). Emission lines *λ* = 178.290 nm, *λ* = 328.06 nm, and *λ* = 242.79 nm were used to determine P, Ag, and Au, respectively. A control sample undergoing the same experimental procedure without addition of the complex was also analyzed and indicated the absence of Ag and Au as possible contaminants. Calibration was carried out by preparing five multi-element standard solutions, containing Ag and P in the concentration range 0–1 mg L^−1^ for Ag and Au and 0–10 mg L^−1^ for P (ppm). Standard solutions were prepared by diluting Ag, Au, and P (Spectrascan standards from Teknolab) stock solutions of 1,000 mg L^−1^with HNO_3_ 2.5% v/v.

#### 2.3.8 Confocal Microscopy Analysis

A2780 cells (5 × 10^4^) were seeded on glass coverslips in 24-well plates and cultured until approximately 50% confluence. Cells were then incubated in the presence of a 75 μM test complex for further 4 h (compound **2**) or 35 min (compound **3**) in standard conditions. After incubation, cells were gently washed with PBS, fixed with 4% formaldehyde at room temperature for 10 min, and permeabilized with 0.1% Triton X-100 in PBS for 5 min. Then, cells were incubated in 7% fetal calf serum in PBS (200 μL) for 30 min in the dark, washed, and stained with Alexa Fluor 488 mouse anti-β-tubulin (BD Pharmingen) for 1 h at room temperature. After washing with PBS, the coverslips were mounted on glass slides by using Mowiol 40–88 (Sigma, St Louis, MO) added with 0.067 μg/μL PI. Images were acquired through ×60 CFI Plan Apochromat Nikon objectives with a Nikon C1 confocal microscope and finally analyzed using NIS Elements software (Nikon Instruments, Florence, Italy), NIH ImageJ, and Adobe Photoshop CS4 version 11.0.2.

## 3 Results

### 3.1 Synthesis and Characterization of the Compounds **1**–**3**

**SCHEME 1:**
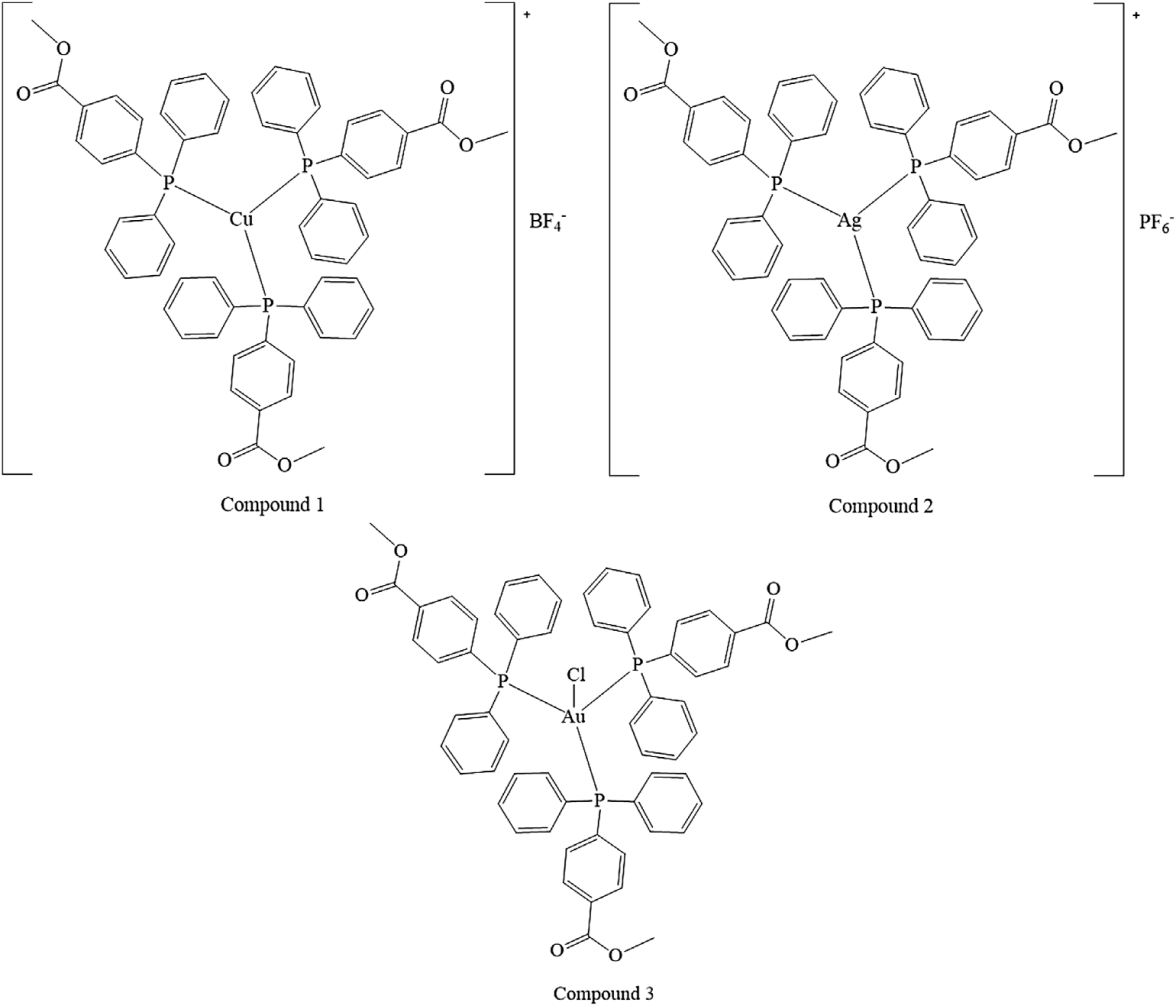
Molecular structures of the compounds 1–3.

Coinage metals feature the anticancer activity of metal-based drugs (Santini et al., 2011; Jakob et al., 2021), triggering the cytotoxic effects and addressing the main paths of the mechanism of action (Boros et al., 2020). The preparation of a homolog series of coordination compounds using a non-cytotoxic luminescent ligand was approached in this work, affording a series of polycoordinated complexes **1**–**3** displaying tri- or tetracoordinate environments in the solid-state with different counterions to avoid solubility concerns. Compounds **1** and **2** present the BF_4_ and PF_6_, respectively, which are coordinatively insignificant in solution, while compound **3** displays the chloride as a counterion, and any attempt to substitute it with less coordinating anions afforded the dissociation to the free ligand and the bis-phosphane gold compound. The ligand L^OMe^ was prepared by using MeOH in the reaction with 4-diphenylphosphine benzoic acid to obtain the corresponding ester (Pucciarelli et al., 2019). Compound **2** was prepared by the direct reaction of the silver salt and the ligand L^OMe^ in the stoichiometric mole ratio 1:3, while derivatives **1** and **3** needed an additional mole of ligand to provide the reduction of Cu(II) to Cu(I) for the former and to obtain a better yield for the latter. The analytical and spectroscopic characterizations of the obtained microcrystalline solids support the molecular structures described in Scheme 1.

The elemental analyses exhibit a good agreement with the proposed structures. The formation in rather high yields of tris-phosphane complexes, ranging between 70 and 90%, highlighting that, despite the steric similarity, the introduction of an ester group in the para position of one phenyl of the Ph_3_P moiety lowers the sigma-donating properties of the phosphane leading to poly-phosphane complexes. The ^31^P NMR chemical shifts in CDCl_3_ display values of −4.73 and –4.68 ppm for PPh_3_ and 4-diphenylphosphane benzoic acid, respectively (Supplementary Table S1, supporting information), while the corresponding methyl ester of the 4-diphenylphosphane benzoic acid is shifted to –5.01 in CDCl_3_, slightly more shielded than that of the PPh_3_ ligand. Upon coordination, the ^31^P NMR signals are shifted to higher frequencies recording a change of chemical shifts, Δδ, that is +5.31 ppm for the Cu complex **1**, about +10.50 ppm for the silver complex **2**, and almost +35 ppm in the case of the gold compound **3** in CDCl_3_ (Supplementary Table S1; Supplementary Figure S1, supporting information). These Δδs are indicative of the coordination to the metal cations, remarking somewhat stronger bonds for the heavier metals of the triads. Nevertheless, all the ^31^P NMR chemical shifts of compounds **1**, **2**, and **3** fall in the same range reported in the literature for similar poly-phosphane compounds (Camalli and Caruso, 1987; Gimeno and Laguna, 1997). The broadness of the signals of compounds **1** and **2** indicates dynamic behavior in solution, which has been largely debated in the literature for copper, silver (Muetterties and Alegranti, 1972; Camalli and Caruso, 1987), and also for gold compounds (King et al., 1992; Hamel et al., 2002; Gimeno and Laguna, 1997; Colburn et al., 1979). The behaviors in the CH_3_OH solution were also studied by the ESI mass spectrometry. The results of the mass spectra discriminate the copper complex **1** from complexes **2** and **3**; for this latter, the corresponding bis-phosphane metal cations were the only positive ions detected in methanol solutions (see Supplementary Figure S2, supporting information), while for complex **1** also, the presence of the tris-phosphane copper ion was detected at low intensity. Further structural evidence in the solid-state comes from IR spectroscopy. The BF_4_ and the PF_6_ anions are shown to be free ions in the solid-state given the symmetric band centered at 1,090 cm^−1^ and 837 cm^−1^ for compounds **1** and **2**, respectively (Beck and Sünkel, 1988). Additional confirmations at the metal core coordination sphere are shown by the strong absorptions at around 1,100 cm^−1^ due to the P_quaternary_ –C stretching mode in all the complexes and by the fact that the ester carbonyl stretching mode displays an intense band at 1719 cm^−1^ for the free ligand, L^OMe^, which is only slightly redshifted at 1721, 1722, and 1720 cm^−1^ for compounds **1**, **2**, and **3**, respectively (see the experimental part and Supplementary Table S1, supporting information). Given the low redshifts, we can rule out the coordination of the ester groups of L^OMe^ in all the complexes, even the silver and the copper ions, which might be favorite according to the Pearson HSAB theory (Pearson, 2002). Other additional information comes from the analysis of the IR in the low range of energies (see Supplementary Figures S3, S4, supporting information). In compound **3**, the Au–Cl bond stretching vibrational mode appears 5 cm^−1^ blue-shifted if compared to Ph_3_PAuCl, with bands at 332 cm^−1^ and a shoulder at 326 cm^−1^ for (L^OMe^)_3_AuCl and at 328 cm^−1^ and 321 cm^−1^ for Ph_3_PAuCl. The two bands centered at about 329 cm^−1^ are likely due to the Au^35^Cl and Au^37^Cl stretching modes (O’Connor et al., 2016), respectively, and they confirm the presence of a bond between the gold center and the chloride ion in (L^OMe^)_3_AuCl to form a tetracoordinate complex in the solid-state (Hamel et al., 2002). The ligand and the **1**–**3** complexes herein considered are strongly emissive both in the visible solid and in solution state (Supplementary Figures S5–S7, supporting information). The emission spectra of the three compounds and the free ligand in the solid-state upon excitation at 310 nm display maxima that are blue-shifted for compounds **1** and **2** and redshifted for compound **3** with respect to the intense emission of free ligand centered at 485 nm (Supplementary Figures S5, S6, supporting information). The emission may be attributed to ligand centered electronic transitions for all the compounds with emission maxima ranging between 430 and 525 nm; for example, upon coordination to gold, the emission maximum at 525 nm in the solid-state is redshifted at 500 nm in the HEPES/methanol solution (Supplementary Figure S7, supporting information); the bis-phosphane gold compound is not emissive, as discussed in the literature and experimentally observed (Pucciarelli et al., 2019; King et al., 1992).

### 3.2 Biological Studies

#### 3.2.1 Antiproliferative Activity

The antiproliferative effect of the tris-arylphosphane ligand and complexes **1**–**3** was evaluated on a panel of human tumor cell lines: HT-29 (colorectal adenocarcinoma), H1975 (non-small-cell lung cancer), MDA-MB-231 (triple-negative breast cancer), and the cisplatin-sensitive and -resistant cell line pair, A2780 and A2780cis (ovarian carcinoma). Cisplatin, the well-known metal-based drug, was reported as reference. The results are shown in Table 1 as GI_50_ values, that is, the concentration of the compound inducing a reduction of 50% in cell number.

**TABLE 1:** Effect of tris-arylphosphane ligand and complexes **1**–**3** on cell growth. Results are expressed as GI_50_ values ± SD of at least three independent experiments in duplicate. Cisplatin is reported as reference.

	GI_50_ (μM)				
Compounds	A2780	A2780cis	HT29	H1975	MDA-MB-231
**Ligand, L^MeO^**	>20	>20	>20	>20	>20
**1**	1.78 ± 0.15	4.40 ± 0.18	4.80 ± 0.50	9.10 ± 0.75	8.47 ± 0.76
**2**	0.17 ± 0.07	0.94 ± 0.10	2.98 ± 0.40	2.07 ± 0.23	3.46 ± 0.30
**3**	0.17 ± 0.03	4.68 ± 0.86	8.17 ± 0.50	2.17 ± 0.15	11.45 ± 0.60
**Cisplatin**	1.08 ± 0.35	4.50 ± 0.88	3.02 ± 0.47	3.50 ± 0.56	50.49 ± 2.0^a^

a Gambini et al.(2018).

**FIGURE 1:**
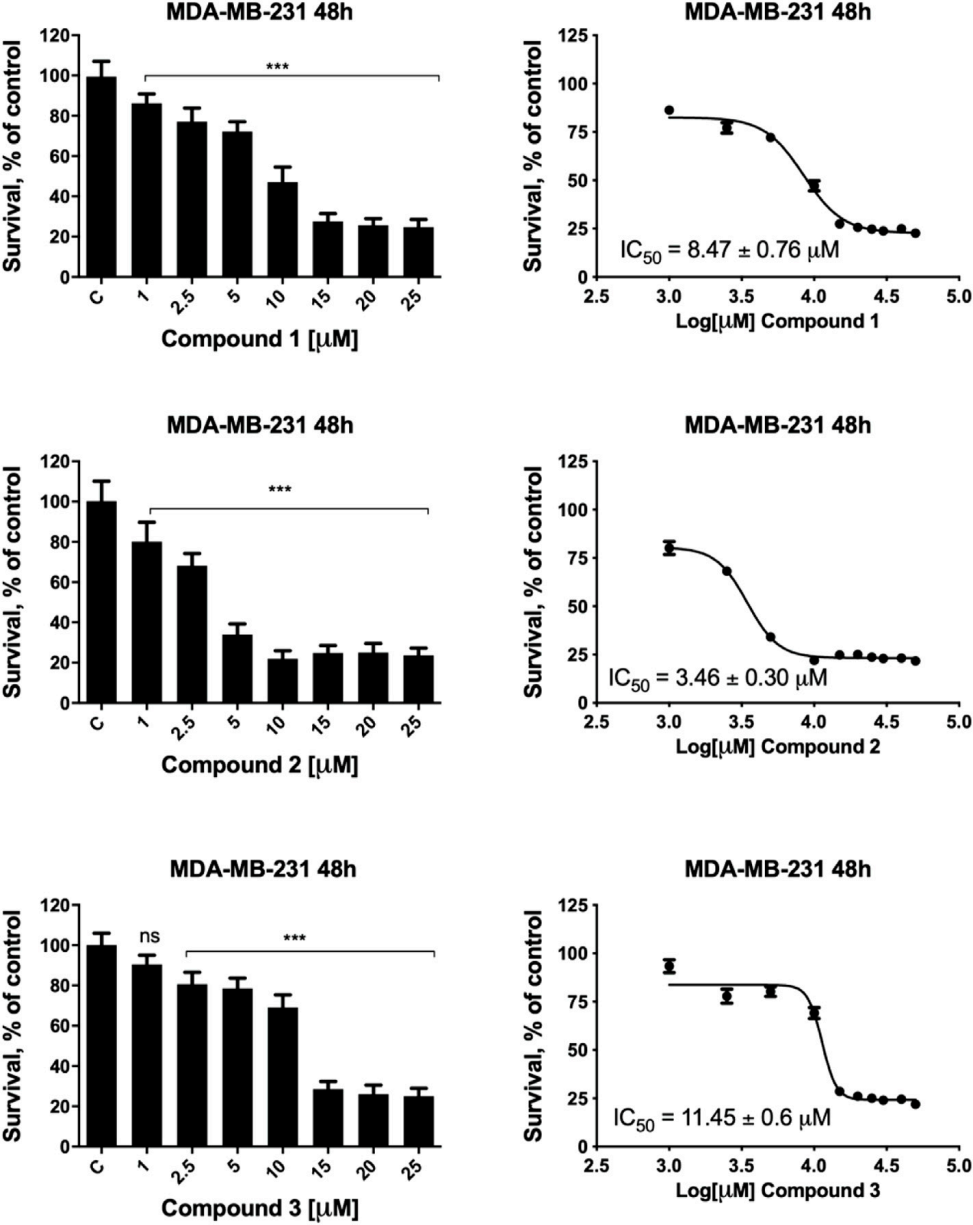
MDA-MB-231 cell viability after treatment with compounds **1**–**3**. MDA-MB-231 cells were incubated in the presence of vehicle or increasing concentrations of compounds **1**–**3** for 48 h, and cell viability was determined by the MTT assay. The results are expressed as percentage of living cells with respect to control (vehicle alone). Columns, mean of three separate experiments, wherein each treatment was repeated in six wells; bars, SD. *p ≤ 0.0332; ***p ≤ 0.0002; ****p ≤ 0.0001, one-way ANOVA followed by Dunnett’s multiple comparison test. GI_50_ values were calculated by fitting the concentration-effect curve data obtained in the three experiments with the sigmoid-E_max_ model using nonlinear regression, weighted by the reciprocal of the square of the predicted effect.

The metal complexes **1**–**3** exert a significant antiproliferative effect on all considered cell lines, with GI_50_ values ranging from 0.17 to 11.45 μM. Complex **2** is the most cytotoxic, and its effectiveness is of particular interest in female cancer cells, MDA-MB-231, A2780, and A2780cis. MDA-MB-231 is a human triple-negative breast cancer cell line. Triple-negative breast cancer is a particularly aggressive malignancy associated with poor prognosis. Treatment with platinum complexes represents one of the most used therapeutic strategies for its management. Nevertheless, notwithstanding it responds to chemotherapy better than other breast cancers, it remains a generally incurable disease. Interestingly, complex **2** is very active in MDA-MB-231 cells, showing a GI_50_ value 14.6 times lower than that obtained for cisplatin. In Figure 1, the plots of the percentage of living cells with respect to control after 48 h are reported for compounds **1**–**3**, and in Supplementary Figure S8, the plots relative to 24 h of treatment are also shown.

A remarkable cytotoxic ability of complex **2** is also observed in ovarian carcinoma cells. In fact, the GI_50_ value decreases about 6 times in the parental A2780 cell line and about 4.7 times in the cisplatin-resistant A2780cis cells, in comparison to the reference drug. This result is very interesting because ovarian cancer is one of the most common gynecologic cancer and is characterized by a high mortality rate. Finally, it is possible to underline that the tris-arylphosphane ligand herein considered is ineffective up to 20 μM concentration on all the tested cell lines. Attempting to draw a comparison between the cytotoxicity of compounds **1**–**3** (Table 1) and previous results on this triad of elements (Jakob et al., 2021; Santini et al., 2011), it is possible only to underline the essential role played by the metals in triggering a synergic action between the metal and the ligand.

#### 3.2.2 Apoptosis and Cell Cycle Analysis

**FIGURE 2:**
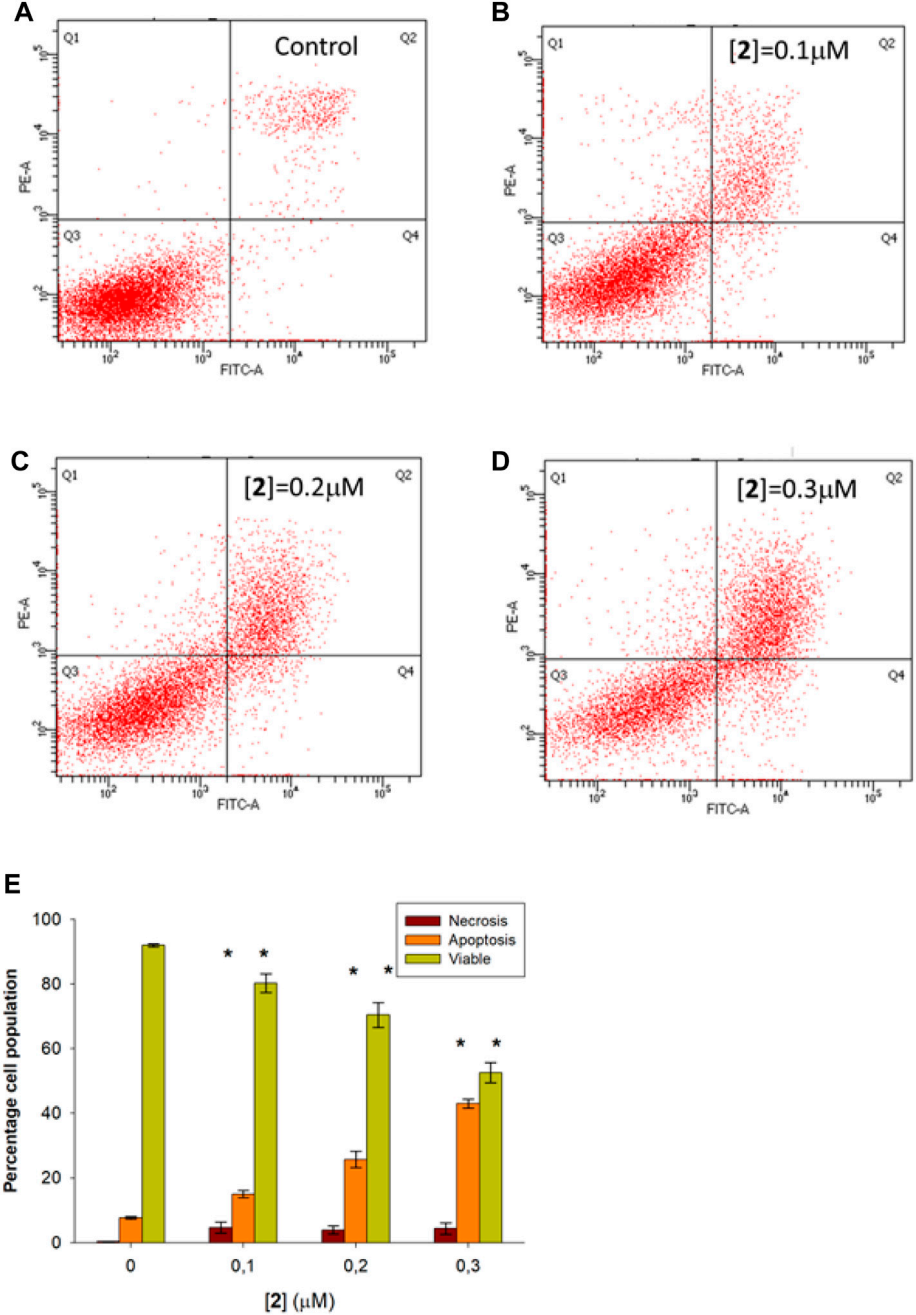
Flow cytometric analysis of apoptosis in A2780 cells treated with **2**. A2780 cells were incubated for 48 h with compound **2** at indicated concentrations and analyzed by flow cytometer after staining with FITC-conjugated Annexin V and PI. The dot plots show a representative experiment for untreated (control, **(A)** and treated cells at 0.1, 0.2, and 0.3 μM concentration (**B–D**, respectively). Bar chart **(E)** represents the percentage of viable (Q3), early plus late apoptosis (Q4+Q2), and necrotic (Q1) cells as a mean ± SD of three independent experiments in duplicate with *p, 0.05 (sample vs*.* control).

Based on the remarkable antiproliferative activity exerted by complex **2** in all cancer cells and, in particular, in ovarian carcinoma, we investigated the intracellular phenomena occurring in A2780 treated with **2** to highlight the possible intracellular targets. Preliminarily, we performed flow cytometry experiments to investigate the cell death mechanism. In detail, A2780 cells were incubated with complex **2** at 0.1–0.3 µM for 48 h, stained with Annexin V-FITC and propidium iodide (PI), and then the percentages of viable, apoptotic, and necrotic cells were analyzed. Figure 2 shows the dot plots of a representative experiment (A–D) and the average mean values of the performed experiments (E). The obtained results indicate that the treatment with **2** promotes the apoptotic process in a dose-dependent fashion. Indeed, the percentage of total (early plus late) apoptotic cells increases from about 7.9% in the untreated condition (control) to about 15.8%, 27.5%, and 44.0% at 0.1, 0.2, and 0.3 µM test complexes, respectively. Otherwise, the necrotic process does not seem to contribute appreciably to the cell death induced by complex **2** because only a slight increase, ranging from about 0.4% (control) to about 5.6% at the higher considered concentration (0.3 µM), is observed.

**FIGURE 3:**
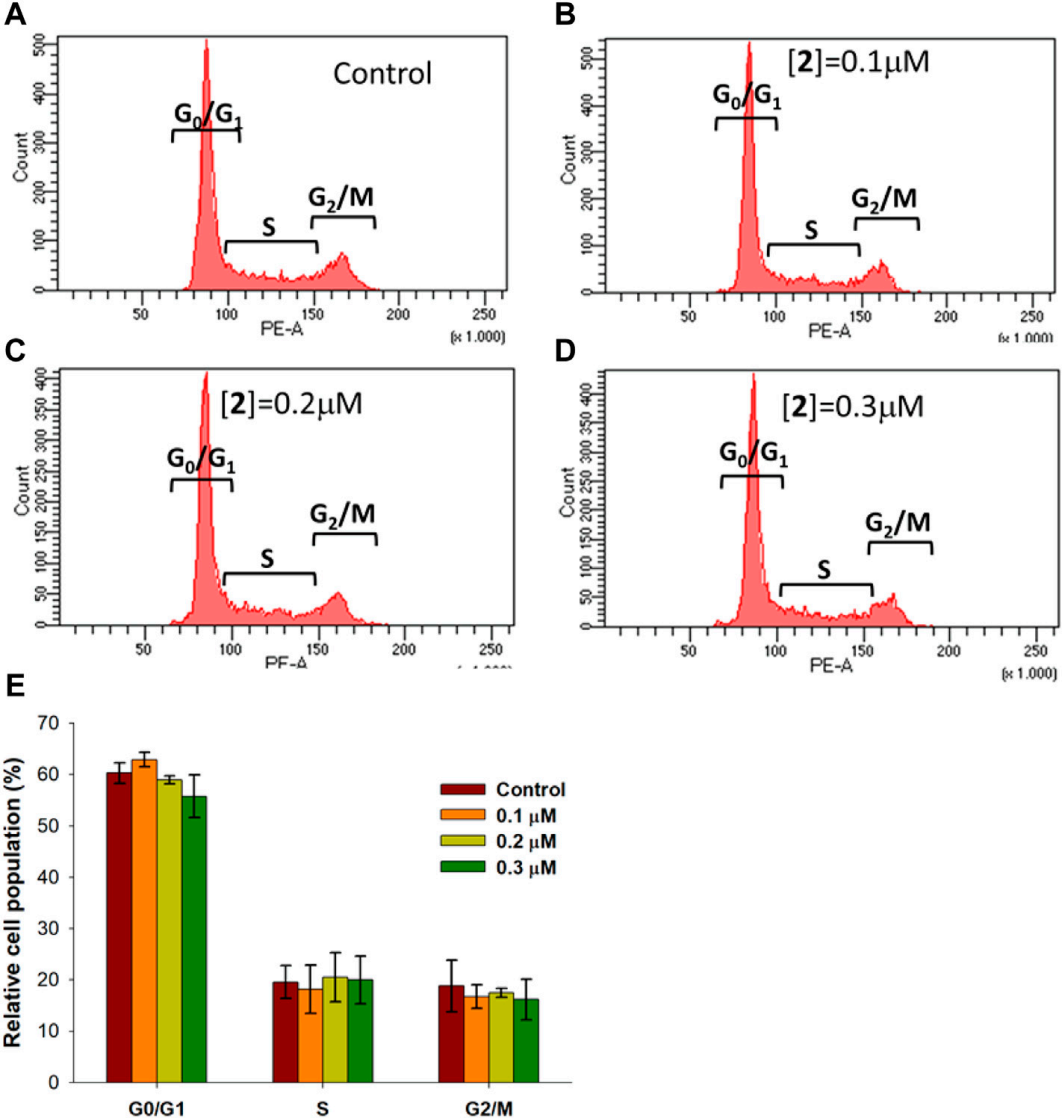
Cell cycle distribution in A2780 cells treated with **2**. A2780 cells were incubated for 48 h with compound **2** at indicated concentrations and analyzed by flow cytometer after staining with PI. The dot plots show a representative experiment for untreated cells (control) **(A)** and cells treated with **2** at 0.1, 0.2, and 0.3 μM concentrations (**B–D**, respectively). Bar chart **(E)** represents the percentage of cells in each cell phase. Data are expressed as the mean ± SD from five independent experiments. No significant difference was found between the control sample (*p* > 0.05) and compound **2** at different concentrations for all cell cycle phases.

A number of metal complexes can activate cell cycle checkpoints, leading to cycle arrest and cell death (Gałczyńska et al., 2020). Cytofluorimetric analysis performed on A2780 cells treated with **2** for 48 h at 0.1, 0.2, and 0.3 μM and stained with PI allowed us to investigate the effect of the complex on cell cycle phases. The results, shown in Figure 3, demonstrate the absence of any significant variation in cell cycle distribution between the control condition and treated cells, suggesting for complex **2** the ability to activate the apoptotic pathway without interfering with cell cycle regulation.

#### 3.2.3 Effect on Mitochondria

**FIGURE 4:**
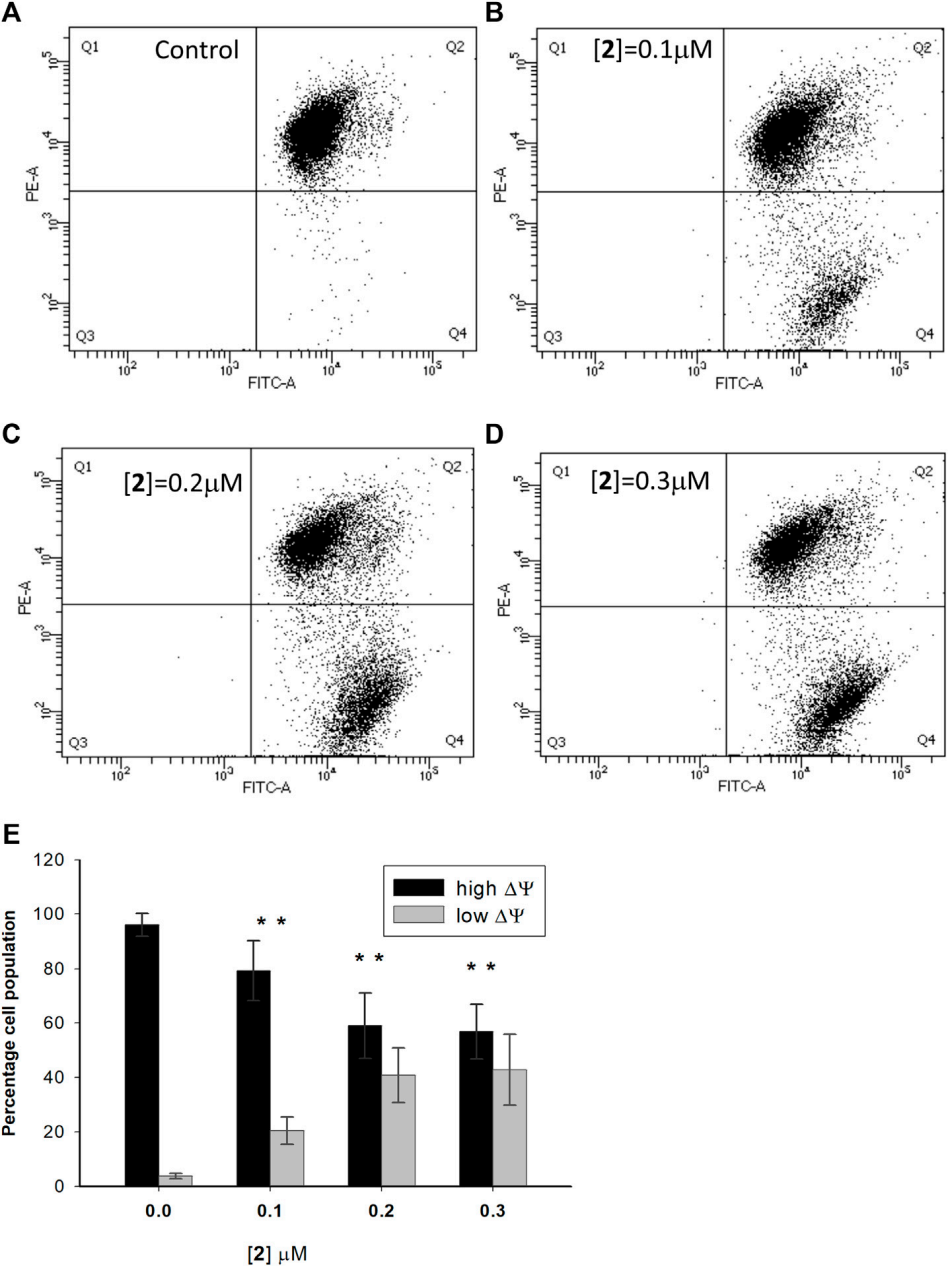
Effect of compound **2** on the mitochondrial membrane potential in A2780 cells. A2780 cells were incubated for 48 h with compound **2** at indicated concentrations and analyzed by a flow cytometer after staining with the cationic fluorescent probe JC-1. The dot plots show a representative experiment for untreated cells (control) **(A)** and cells treated with **2** at 0.1, 0.2, and 0.3 μM concentrations (**B–D**, respectively). The bar chart **(E)** represents the percentage of cell population with high (Q2) and low (Q4) values of mitochondrial transmembrane potential. Data are expressed as the mean ± SD from five independent experiments. *p < 0.05, in comparison to the control sample.

Mitochondria are the mainstay of the intrinsic pathway of apoptosis. This route to cell death is initiated by intracellular stimuli that induce crucial mitochondrial events, such as channel opening, the release of pro-apoptotic factors, changes in the inner membrane permeability, and loss of mitochondrial membrane potential (Kroemer et al., 2007). To investigate the involvement of mitochondria in the apoptotic death induced by **2**, A2780 cells were incubated with the test complex in experimental conditions as in the apoptosis assay and loaded with the cationic fluorescent probe JC-1. At low concentrations, the dye exists as a monomer and emits green fluorescence; at high concentrations, it forms aggregates, yielding red fluorescence. In healthy cells, JC-1 accumulates at a high concentration in mitochondria driven by the inner membrane potential, negative inside, leading to red fluorescent aggregates. In cells undergoing mitochondrial depolarization, JC-1 leaks out from the mitochondria into the cytosol as monomers, and this event is detected as a decrease in red fluorescence. Figure 4A–D show a representative experiment performed in A2780 cells treated with complex **2** and analyzed by a flow cytometer. In Figure 4E, the mean data and statistical analysis from all performed experiments are shown. Test complex induces a significant and dose-dependent mitochondrial membrane depolarization, with an increase of cells with depolarized mitochondria from about 20.6% to about 42.8% at 0.1 and 0.3 µM concentrations of **2**, respectively. It is interesting to note that this behavior is in agreement with the data obtained in the apoptosis assay (see Figure 2), thus suggesting a relationship between the mitochondria impairment and the activation of the apoptotic process and then the ability of complex **2** to induce cell death through the intrinsic apoptotic pathway.

#### 3.2.4 Cellular Redox State

**FIGURE 5:**
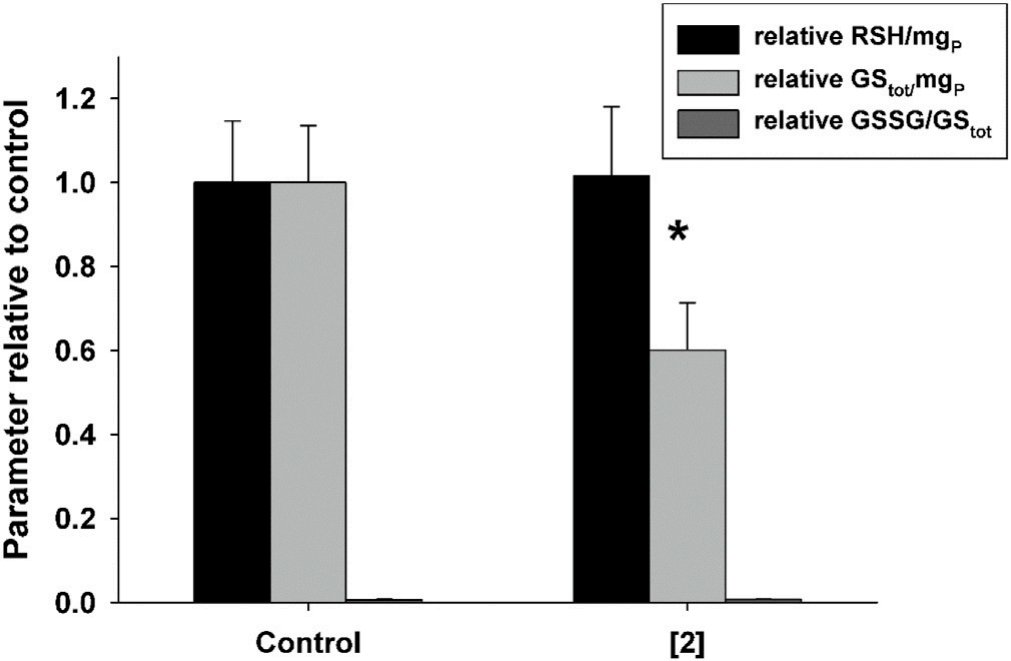
Effect of compound **2** on the total content of thiols (RSH) and glutathione (GStot) and on the GSSG/GS_tot_ ratio in A2780. Cells were treated with 0.2 μM compound **2** for 48 h. Mean values and SD from three independent experiments are shown. *p < 0.05 significant difference in comparison to the control sample.

As compound **2** was found to affect mitochondrial membrane potential, with potential consequences on reactive oxygen species generation and ATP synthesis, its possible effect on the redox state of A2780 cells was evaluated. With this aim, A2780 cells were incubated for 48 h in the presence of compound **2** at 0.2 µM (that is, a value close to GI_50_) and the total thiols (RSH), the reduced glutathione (GSH), and the ratio between oxidized and total glutathione (GSSG/GS_tot_) were determined. Results shown in Figure 5 indicate that compound **2** induces a significant decrease in the total glutathione content (GS_tot_), while it is ineffective on both RSH and GSSG/GS_tot_ ratios, this latter has a very low value both in the absence and in the presence of test compounds (6.1 ± 1.9 × 10^−3^ vs. 7.6 ± 0.2 × 10^−3^, respectively). This datum might be explained by a reduction in the glutathione synthesis process, as a consequence of the depolarization of the mitochondrial membrane. This latter phenomenon indeed could cause the impairment of the oxidative phosphorylation process, leading to a reduction in the ATP intracellular content, essential for the synthesis of the tripeptide glutathione.

#### 3.2.5 Cell Uptake

**FIGURE 6:**
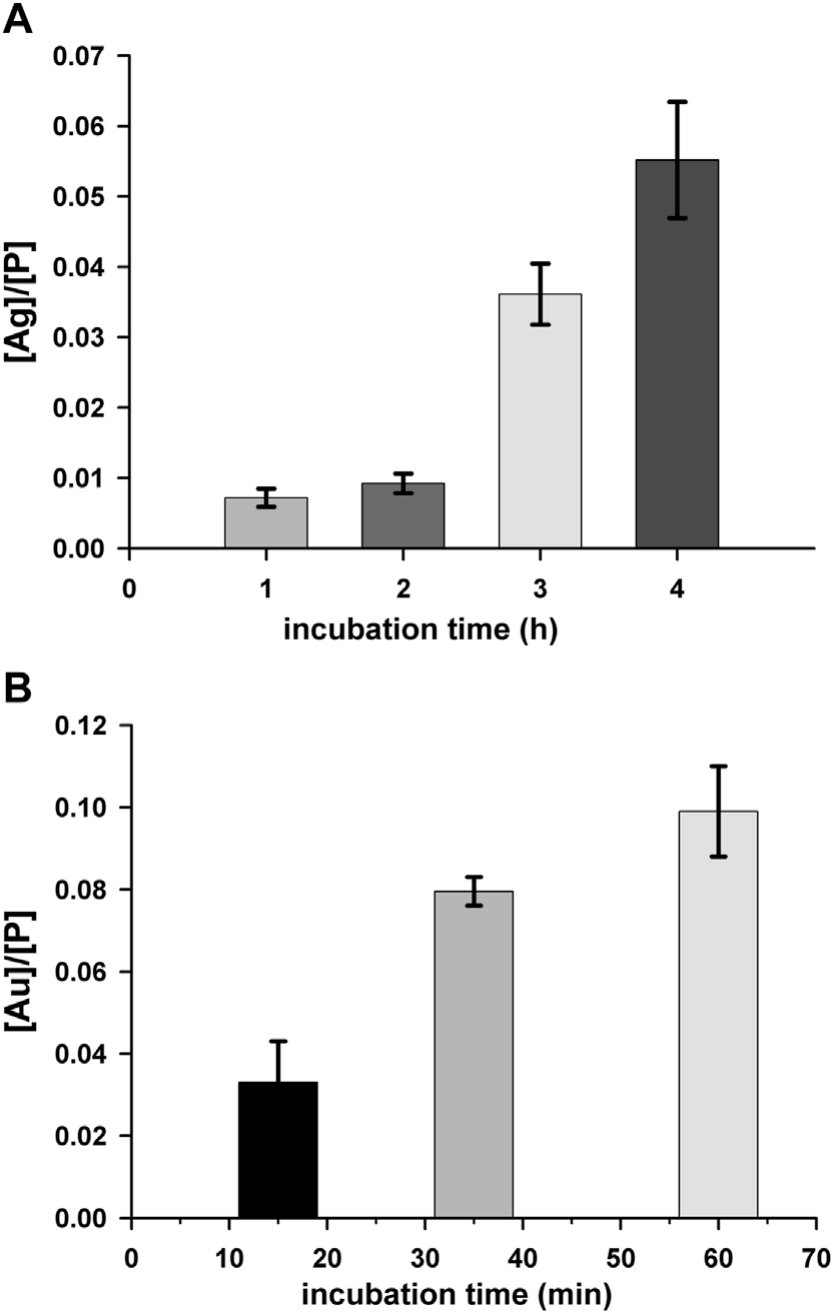
Cell uptake for complex **2 (A)** and **3 (B)**. A2780 cells were incubated with a 75 μM concentration of test complex for different time intervals. Silver (ppb)/phosphorus (ppb) ([Ag]/[P]) and gold (ppb)/phosphorus (ppb) [Au]/[P]) ratios are shown as a function of the incubation time. Mean values ±SD of three independent experiments are reported.

To further deepen the understanding of the biological effects of **2**, the ability of the complex to enter cells was investigated. In detail, we evaluated by ICP-AES the accumulation of **2** in A2780 cells and, by confocal microscopy, the intracellular distribution. In detail, the cell uptake was evaluated by estimating the total amount of Ag, expressed in ppb, in cells treated with a 75 µM test compound for different incubation times (1–4 h). In the samples, the amount of P, in ppb, was also determined, as internal control, according to a previously reported method (Hyeraci et al., 2021). The constant value of ppb of P detected (data not shown) indicates that the viability of treated cells is not compromised in the experimental conditions taken into consideration. Data in Figure 6A show a significant time-dependent increase of the amount of Ag in A2780 cells incubated with **2** demonstrating the capacity of the compound to cross the cell membrane. By considering the chemical structure, it is reasonable to assume that the uptake could be facilitated by the lipophilicity of the tris-phosphane moiety. In this connection, it appeared of interest to assay also the cell uptake of complex **3**, which showed similar cytotoxicity of complex **2** in this cell line. The obtained results (Figure 6B) confirmed an intracellular accumulation also for **3**, whose uptake rate is even faster than that of complex **2**, likely as a consequence of the higher lipophilic character of the neutral gold complex.

**FIGURE 7:**
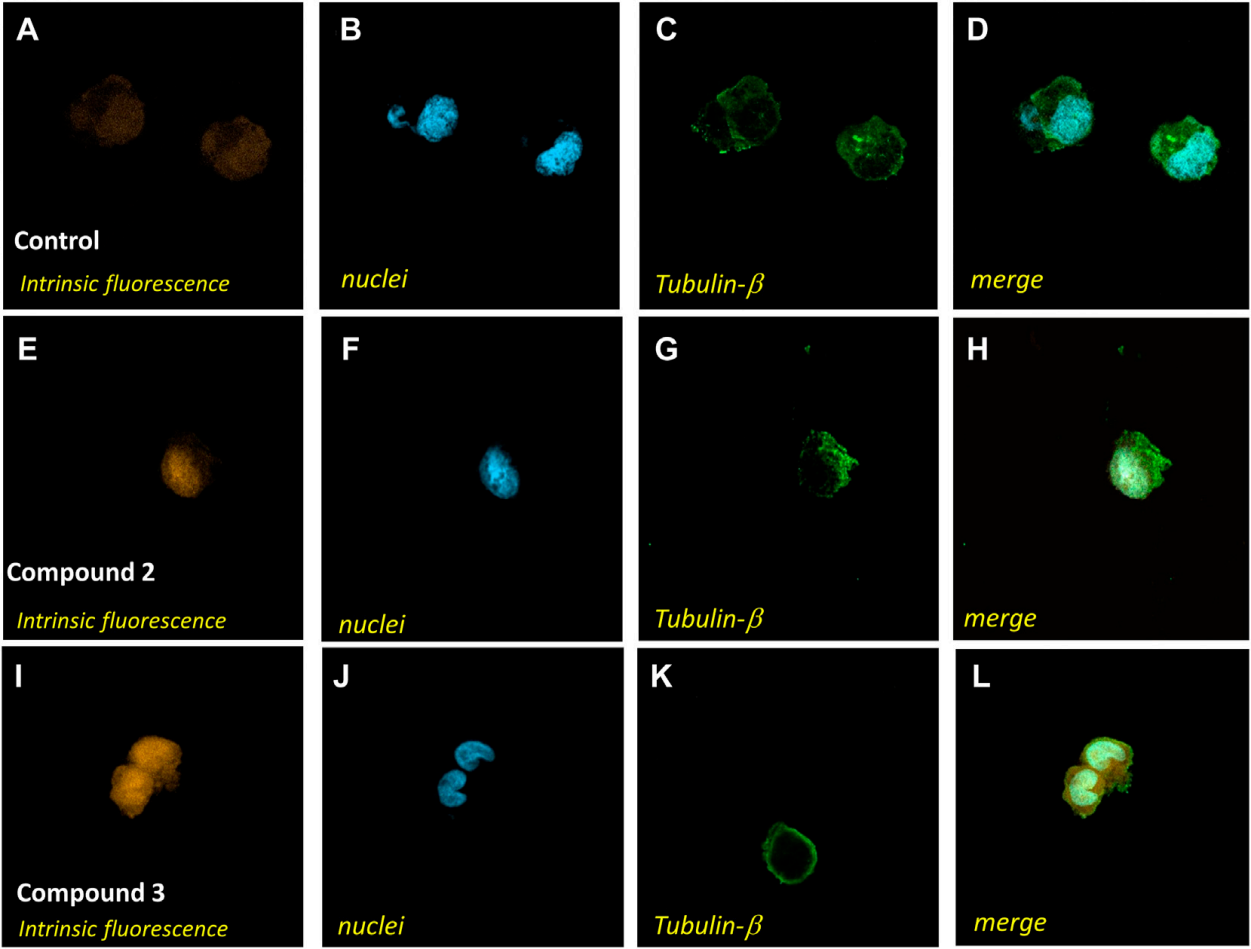
Confocal microscopy analysis of A2780 cells treated with complex **2** or **3**. A2780 cells were incubated in standard conditions **(A–D)**, for 4 h with compound **2** at a 75 μM concentration **(E–H)**, or for 35 min with compound **3** at a 75 μM concentration **(I–L)** and stained with Alexa Fluor 488 Mouse anti-β-tubulin antibody and DAPI to identify the cytoplasmatic compartment **(C,G,K)** and nuclei **(B,F,J)**. Intrinsic fluorescence in **A**, **E**, and **I**

To further demonstrate the ability to cross the cell membrane, we performed confocal microscopy analysis of A2780 cells incubated in standard conditions (Figures 7A–D) in the presence of 75 µM solutions of **2** for 4 h (Figures 7E–H) or in the presence of 75 µM solutions of **3** for 35 min (Figures 7I–L). The collected images support the accumulation of the complexes in cells and show a distribution in the whole cell, confirming a great ability to permeate biological membranes.

## 4 Conclusion

The antiproliferative activity of a homolog tris-phosphane series of complexes obtained with the ligand 4-diphenyl-phosphane methyl ester and coinage metals in the +1 oxidation state has been ascertained on a panel of human tumor cell lines and female cancer cells, such as ovarian carcinoma (A2870 and A2780cis) and breast cancer (MDA-MB-231) cells. The silver tris-phosphane cationic compound **2** is the most effective in inducing cytotoxicity and is more active than cisplatin in the considered cancer cells, with ovarian carcinoma A2780 as the most sensitive to the treatment. Taking into account that the free ligand is ineffective, cytotoxicity assays highlight an interesting empowering effect exerted by the metal center. The higher activity attained for the silver compound **2**, despite the copper and gold homologs, somewhat resembles that observed for an NHC–carbene series whose anticancer activity was ascertained against HeLa and MCF-7 cancer cells (Jakob et al., 2021). Based on these results and considering that few studies have been reported in the literature on anticancer silver phosphine compounds, additional investigations have been led on ovarian cancer cells A2780 after the treatment with the silver compound **2**. The study of the intracellular mechanism of action revealed the activation of the apoptotic pathway with the likely involvement of mitochondria. The cellular accumulation and the distribution of the compound in the whole cell were also demonstrated and compared to the gold homolog.

## Data Availability

The original contributions presented in the study are included in the article/Supplementary Material; further inquiries can be directed to the corresponding authors.
