# Supplementary material for: Coinage Metal Compounds With 4-Methoxy-Diphenylphosphane Benzoate Ligand Inhibit Female Cancer Cell Growth
Source: Front Chem. 2022 Jul 13;10:924584. doi: 10.3389/fchem.2022.924584 (PMC9325969; doi:10.3389/fchem.2022.924584)
Supplement: Supplementary file 1 [file Presentation1.pptx]

## Slide 1
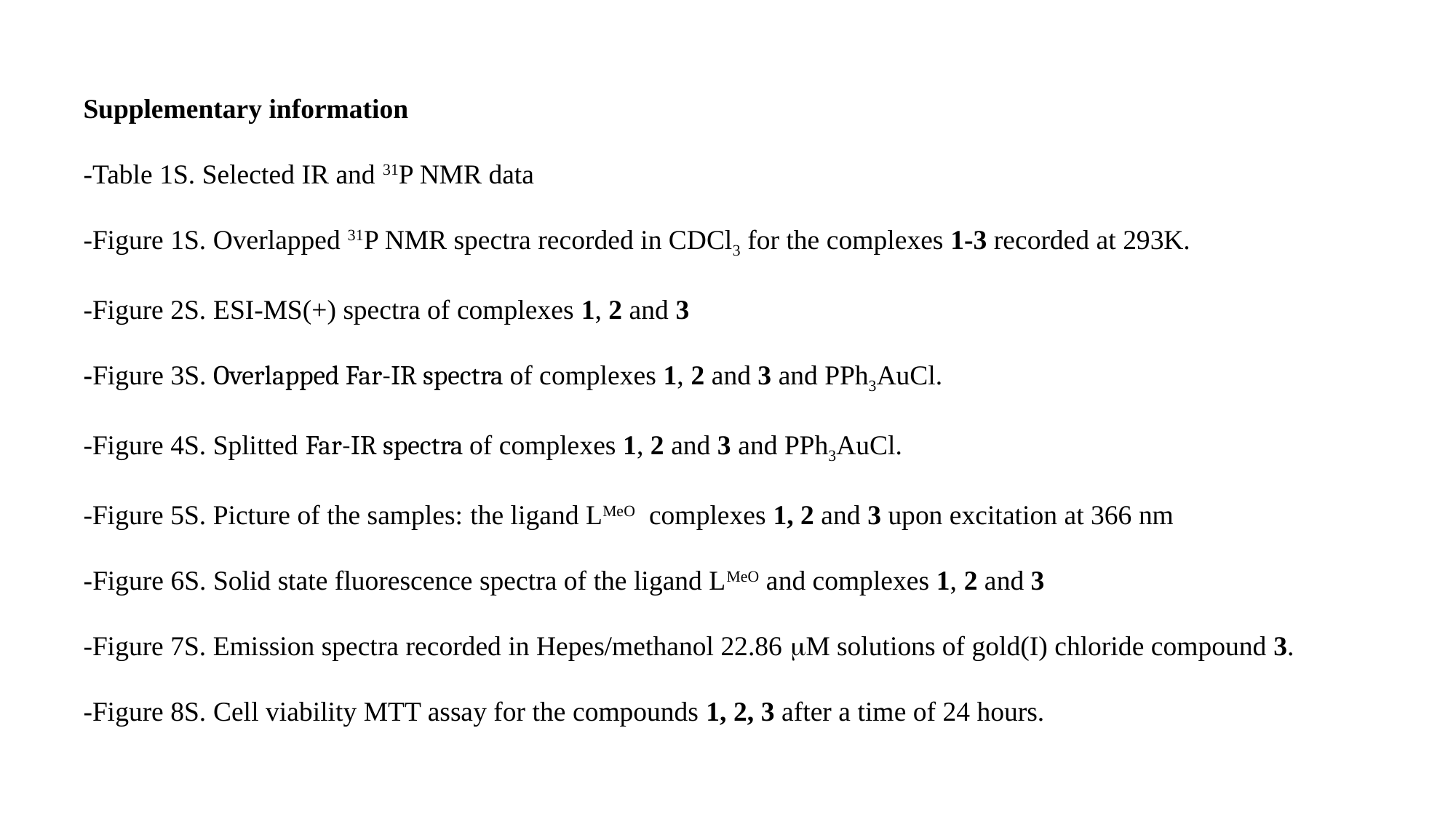

Supplementary information
-Table 1S. Selected IR and 31P NMR data
-Figure 1S. Overlapped 31P NMR spectra recorded in CDCl3 for the complexes 1-3 recorded at 293K.
-Figure 2S. ESI-MS(+) spectra of complexes 1, 2 and 3
-Figure 3S. Overlapped Far-IR spectra of complexes 1, 2 and 3 and PPh3AuCl.
-Figure 4S. Splitted Far-IR spectra of complexes 1, 2 and 3 and PPh3AuCl.
-Figure 5S. Picture of the samples: the ligand LMeO complexes 1, 2 and 3 upon excitation at 366 nm
-Figure 6S. Solid state fluorescence spectra of the ligand LMeO and complexes 1, 2 and 3
-Figure 7S. Emission spectra recorded in Hepes/methanol 22.86 μM solutions of gold(I) chloride compound 3.
-Figure 8S. Cell viability MTT assay for the compounds 1, 2, 3 after a time of 24 hours.

## Slide 2
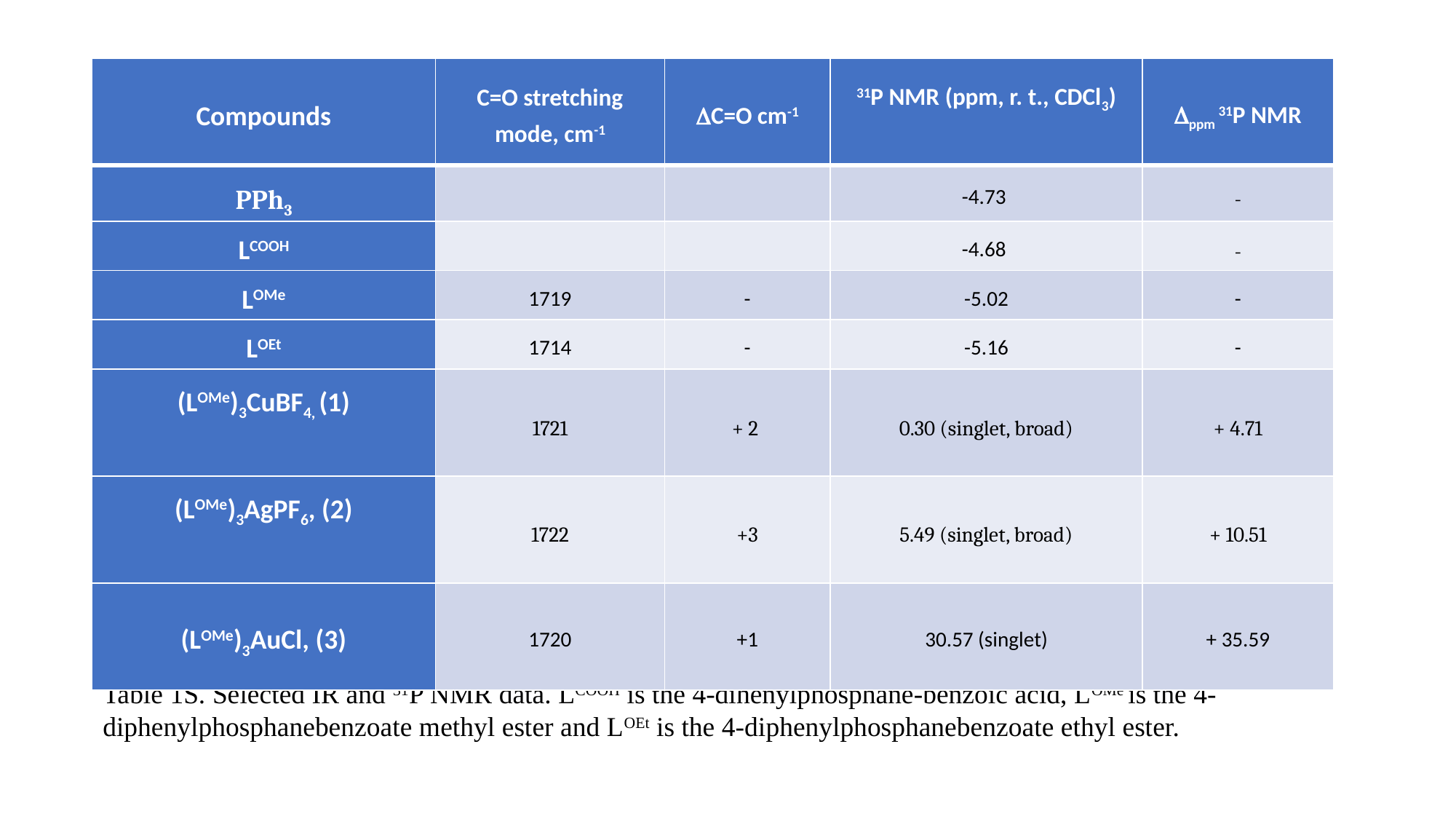

| Compounds | C=O stretching mode, cm-1 | ΔC=O cm-1 | 31P NMR (ppm, r. t., CDCl3) | Δppm 31P NMR |
| --- | --- | --- | --- | --- |
| PPh3 | | | -4.73 | - |
| LCOOH | | | -4.68 | - |
| LOMe | 1719 | - | -5.02 | - |
| LOEt | 1714 | - | -5.16 | - |
| (LOMe)3CuBF4, (1) | 1721 | + 2 | 0.30 (singlet, broad) | + 4.71 |
| (LOMe)3AgPF6, (2) | 1722 | +3 | 5.49 (singlet, broad) | + 10.51 |
| (LOMe)3AuCl, (3) | 1720 | +1 | 30.57 (singlet) | + 35.59 |
Table 1S. Selected IR and 31P NMR data. LCOOH is the 4-dihenylphosphane-benzoic acid, LOMe is the 4-diphenylphosphanebenzoate methyl ester and LOEt is the 4-diphenylphosphanebenzoate ethyl ester.

## Slide 3
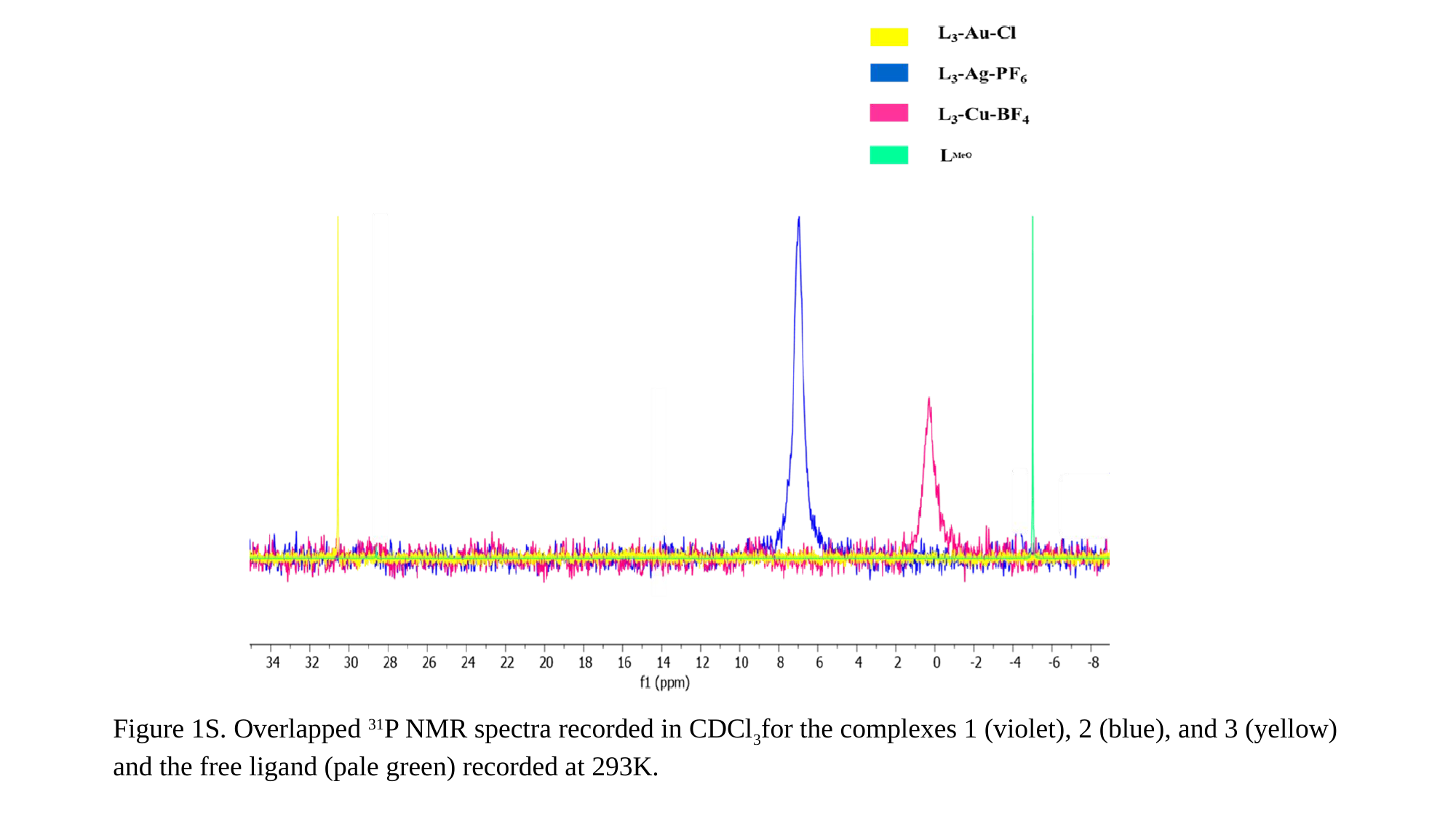

Figure 1S. Overlapped 31P NMR spectra recorded in CDCl3for the complexes 1 (violet), 2 (blue), and 3 (yellow) and the free ligand (pale green) recorded at 293K.

## Slide 4
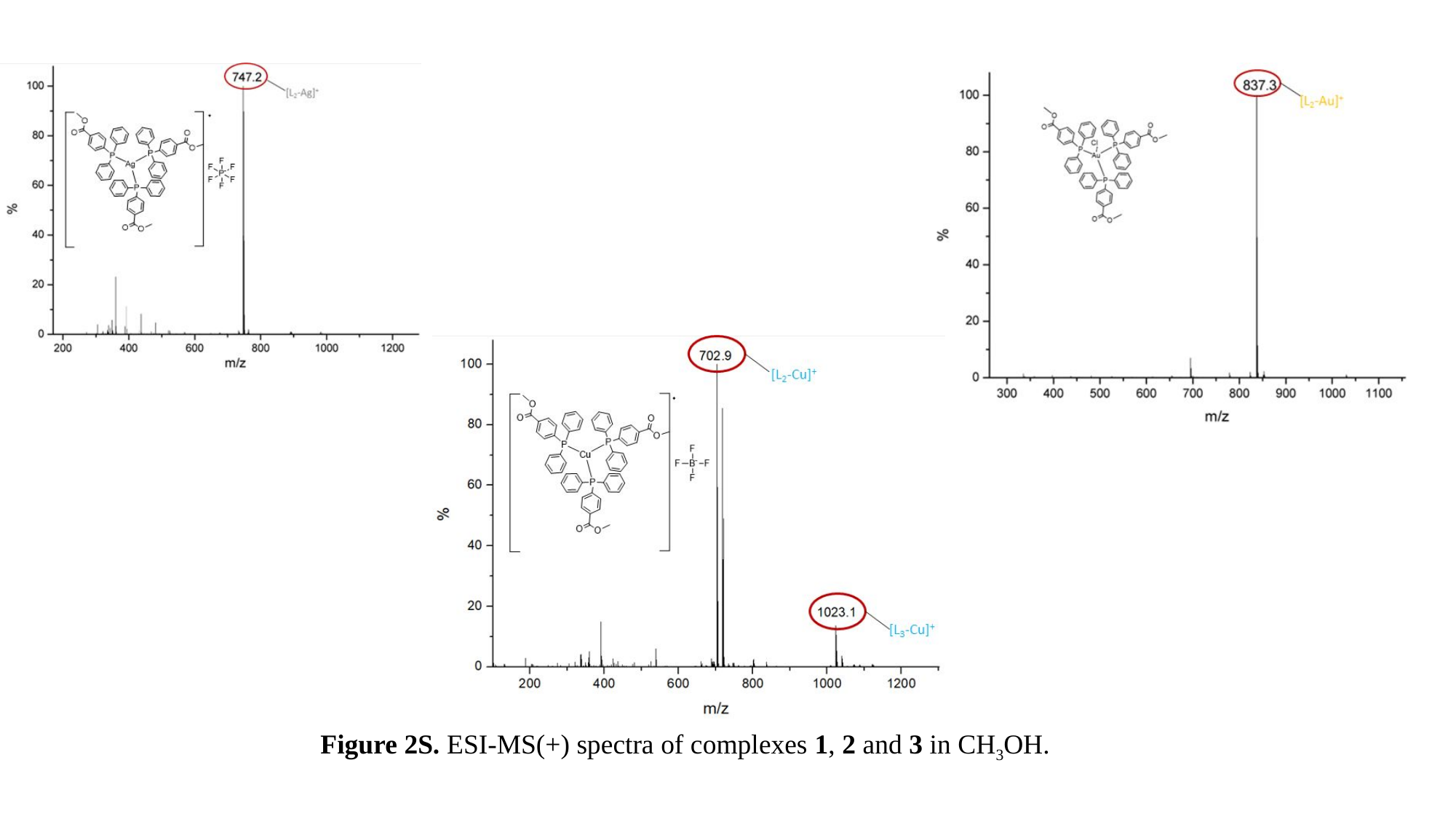

Figure 2S. ESI-MS(+) spectra of complexes 1, 2 and 3 in CH3OH.

## Slide 5
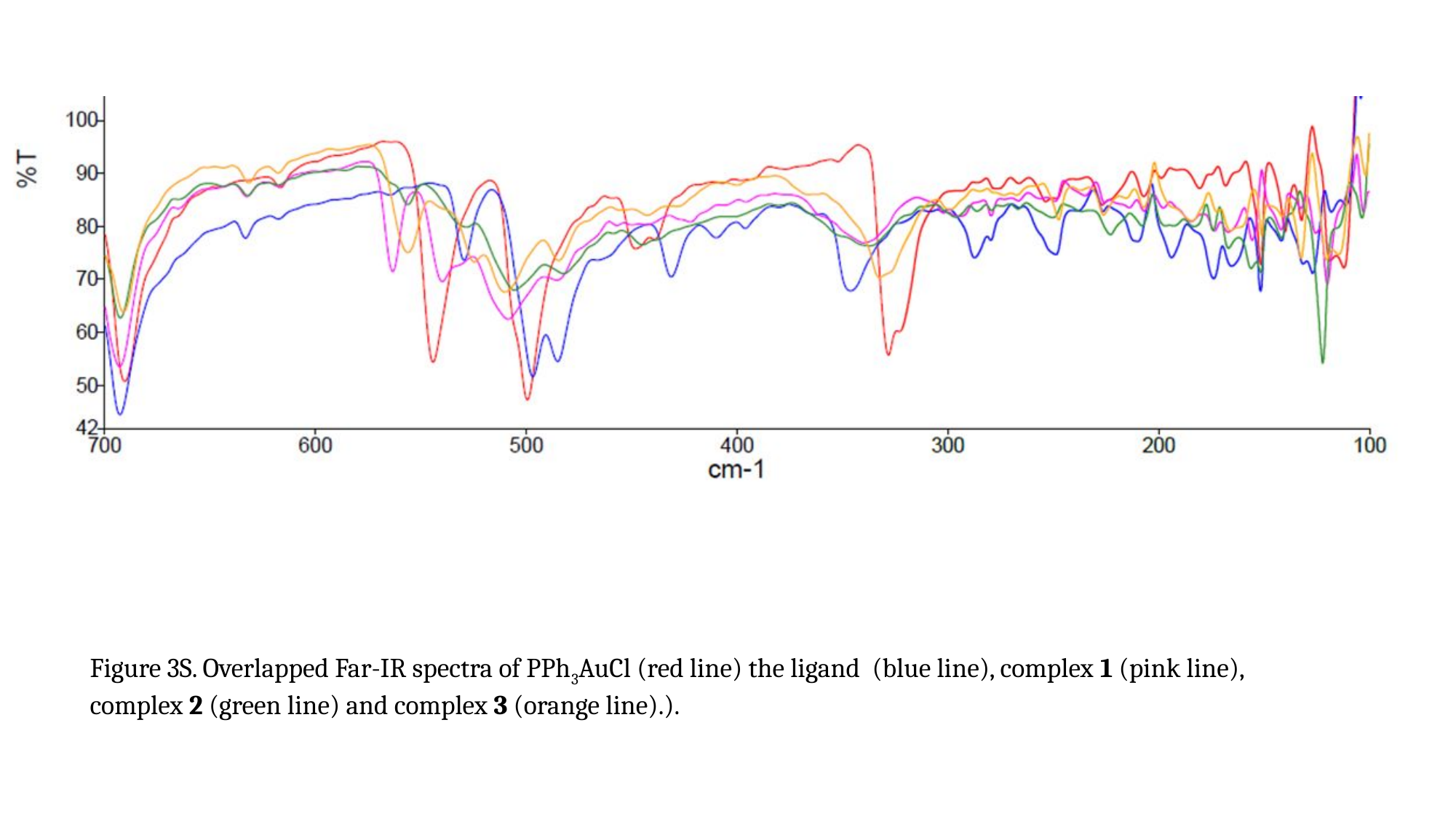

Figure 3S. Overlapped Far-IR spectra of PPh3AuCl (red line) the ligand (blue line), complex 1 (pink line), complex 2 (green line) and complex 3 (orange line).).

## Slide 6
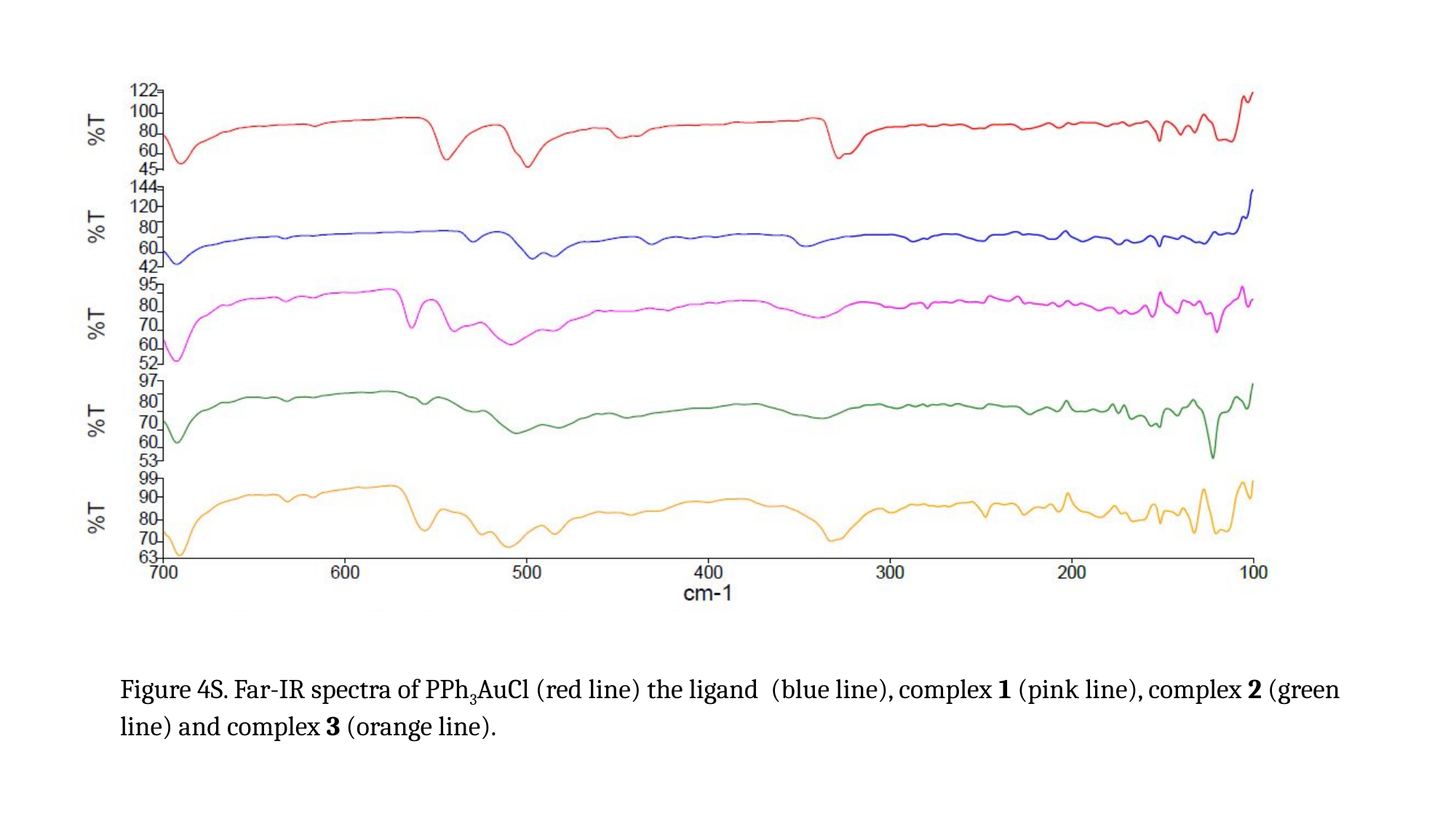

Figure 4S. Far-IR spectra of PPh3AuCl (red line) the ligand (blue line), complex 1 (pink line), complex 2 (green line) and complex 3 (orange line).

## Slide 7
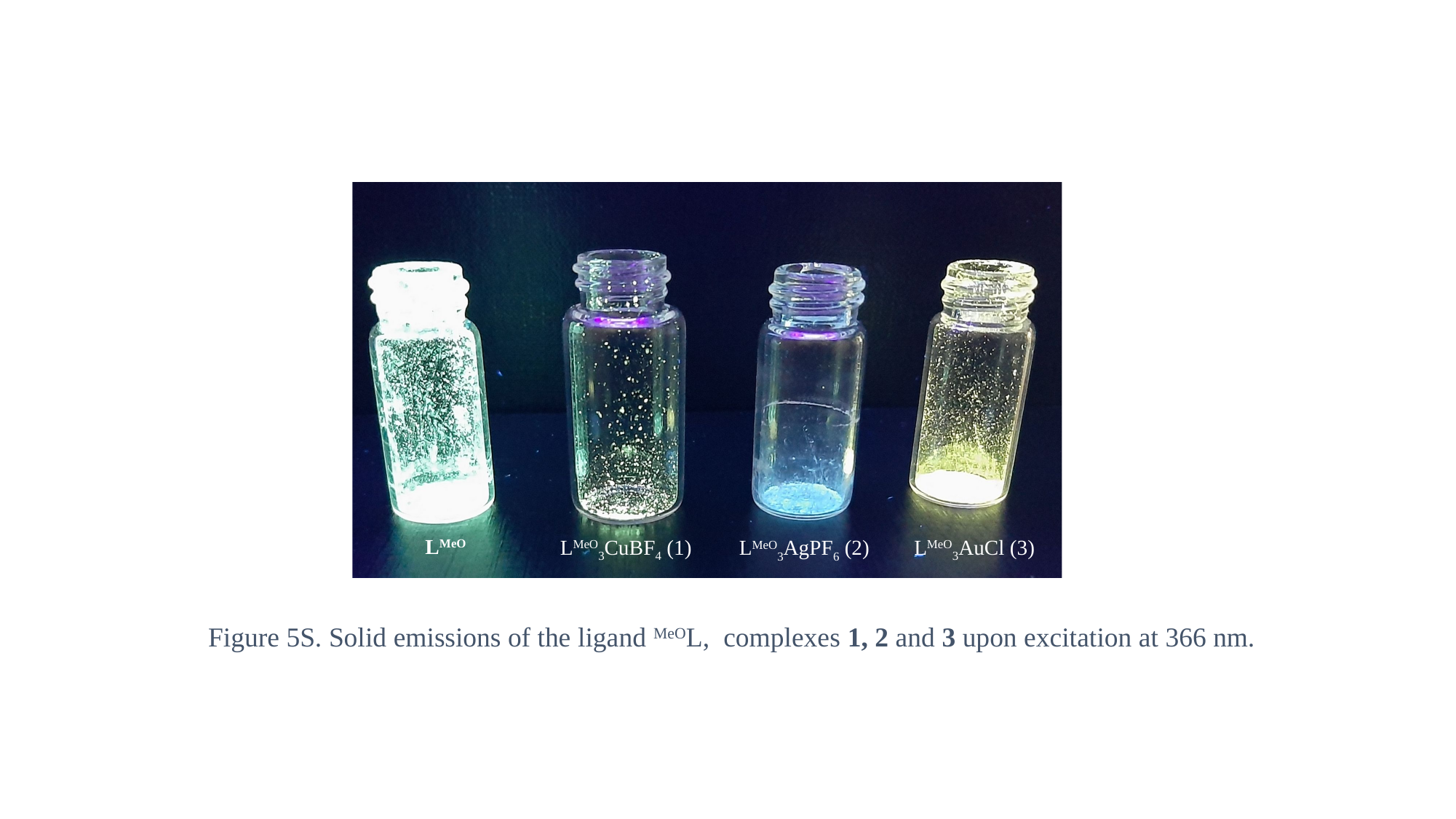

LMeO
LMeO3CuBF4 (1)
LMeO3AuCl (3)
LMeO3AgPF6 (2)
Figure 5S. Solid emissions of the ligand MeOL, complexes 1, 2 and 3 upon excitation at 366 nm.

## Slide 8
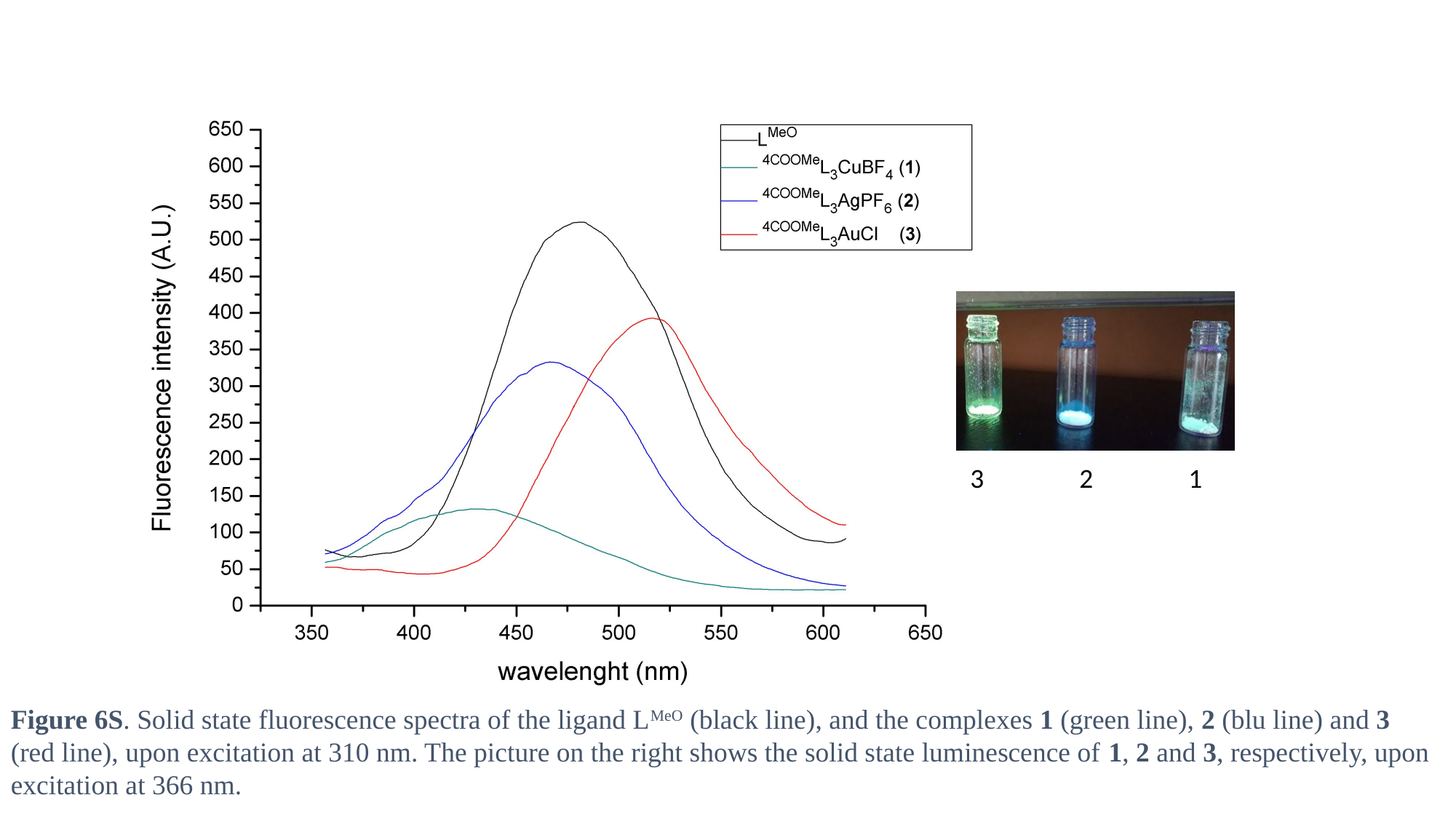

3	2	1
Figure 6S. Solid state fluorescence spectra of the ligand LMeO (black line), and the complexes 1 (green line), 2 (blu line) and 3 (red line), upon excitation at 310 nm. The picture on the right shows the solid state luminescence of 1, 2 and 3, respectively, upon excitation at 366 nm.

## Slide 9
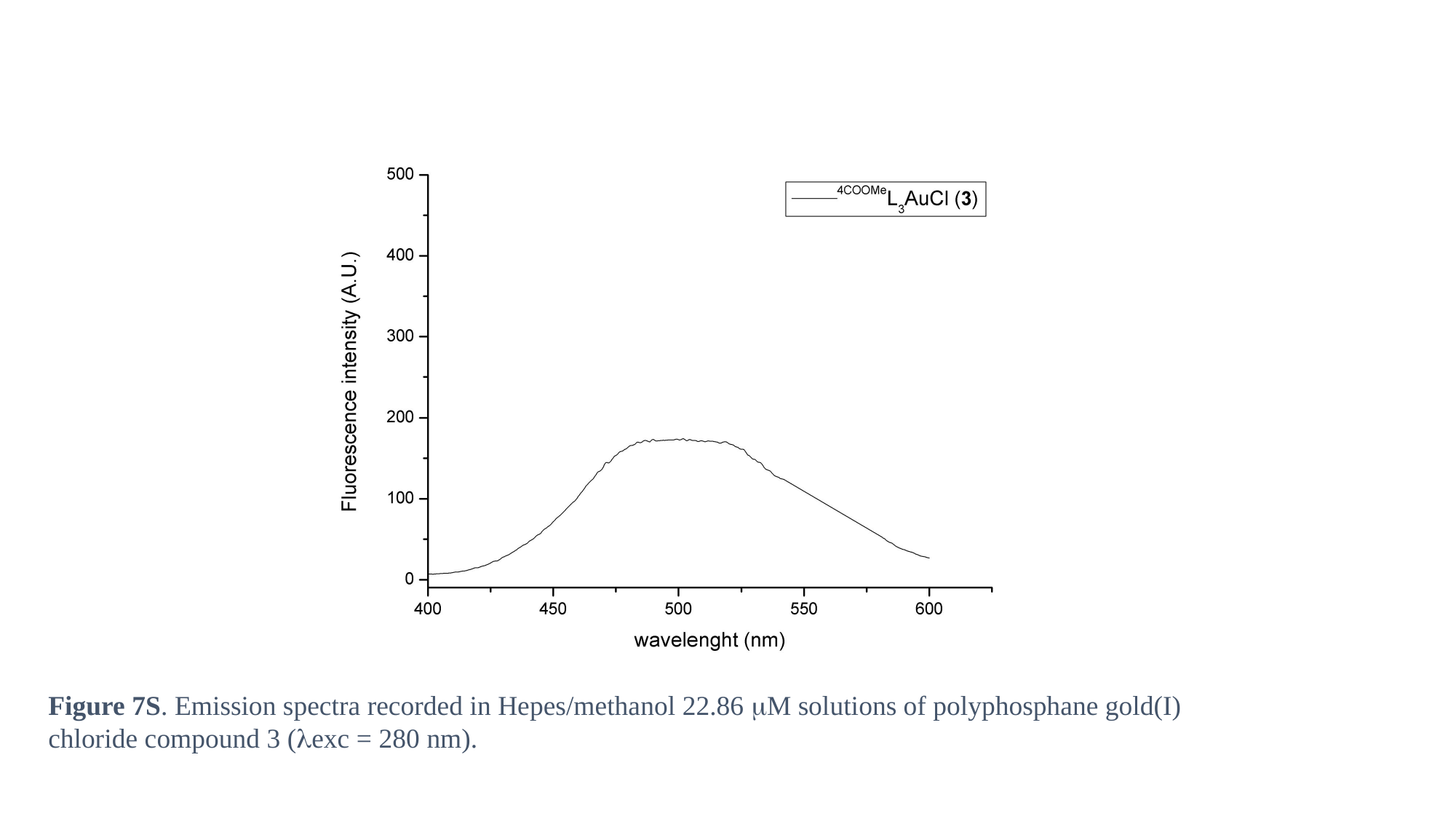

Figure 7S. Emission spectra recorded in Hepes/methanol 22.86 μM solutions of polyphosphane gold(I)
chloride compound 3 (λexc = 280 nm).

## Slide 10
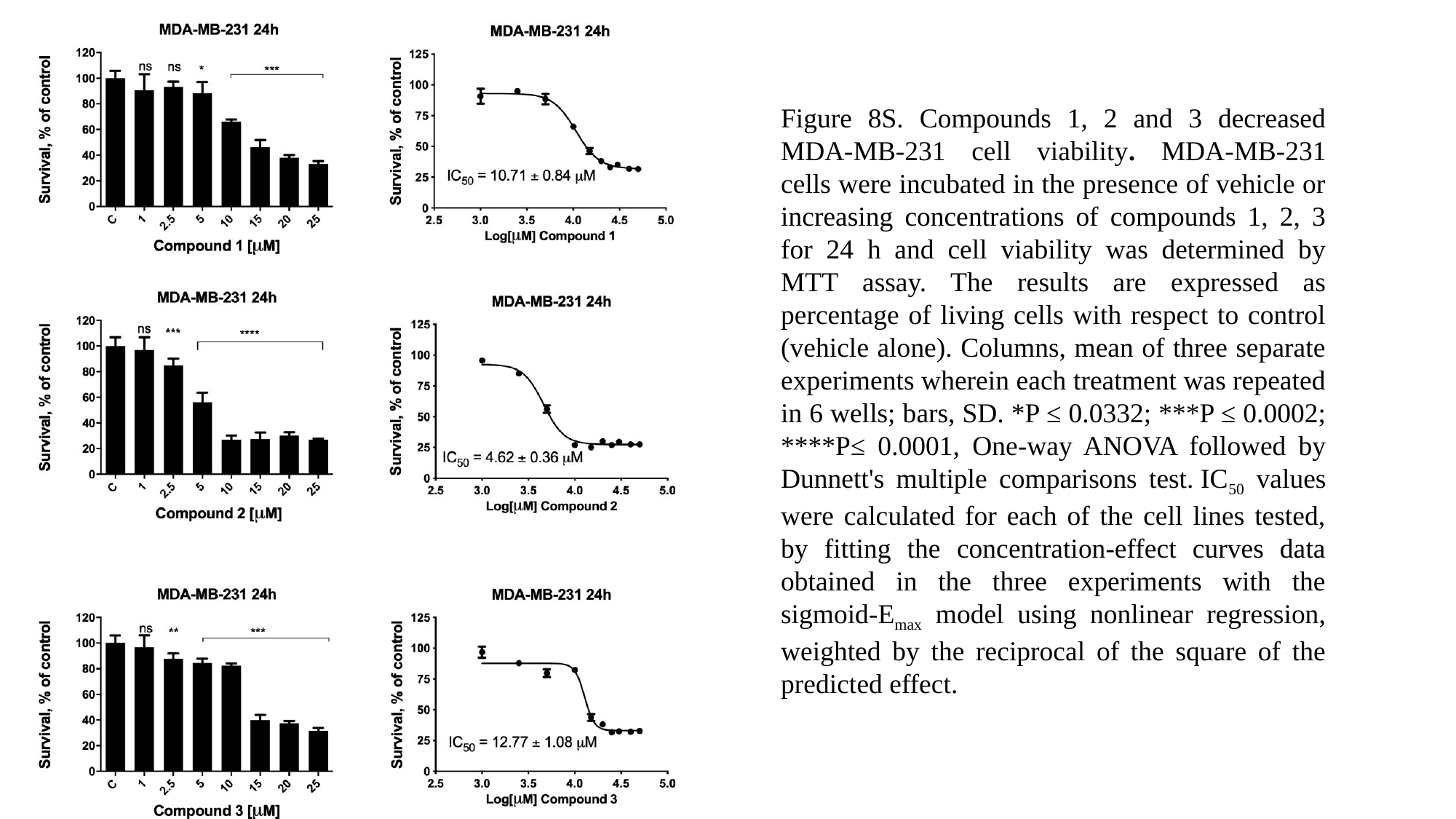

Figure 8S. Compounds 1, 2 and 3 decreased MDA-MB-231 cell viability. MDA-MB-231 cells were incubated in the presence of vehicle or increasing concentrations of compounds 1, 2, 3 for 24 h and cell viability was determined by MTT assay. The results are expressed as percentage of living cells with respect to control (vehicle alone). Columns, mean of three separate experiments wherein each treatment was repeated in 6 wells; bars, SD. *P ≤ 0.0332; ***P ≤ 0.0002; ****P≤ 0.0001, One-way ANOVA followed by Dunnett's multiple comparisons test. IC50 values were calculated for each of the cell lines tested, by fitting the concentration-effect curves data obtained in the three experiments with the sigmoid-Emax model using nonlinear regression, weighted by the reciprocal of the square of the predicted effect.
